# Supplementary material for: Divergent mechanisms regulate conserved cardiopharyngeal development and gene expression in distantly related ascidians
Source: eLife. 2014 Sep 10;3:e03728. doi: 10.7554/eLife.03728 (PMC4356046; doi:10.7554/eLife.03728)
Supplement: Supplementary file 1. — DNA sequences of probes, enhancers, promoters, protein-coding cDNAs, in situ hybridization probe templates, primers, etc used in this study. DOI: http://dx.doi.org/10.7554/eLife.03728.029 [file elife03728s001.docx]

SUPPLEMENT - DNA SEQUENCES

Due to the imprecisions arising from sequencing/assembly mistakes or polymorphisms, the sequences published below are in accordance with the published genome assemblies and may not be identical to the actual sequence of cloned fragments (unless indicated otherwise by the term “VERIFIED SEQUENCE”). “N”s indicate gaps in sequencing.

**TEMPLATES FOR IN SITU HYBRIDIZATION RNA PROBES**

Moocci.Mesp 5’RACE (VERIFIED SEQUENCE):

ATACTAAATTCATCAGTATATATATCATCTATATCTATACAGTGGGTAATAAGTCTTAATAGATATCGGACAAAGTAAAAACTGCATATTGAAGCGTTTGAAAAAAGTTGTTCAATTCAATAGAAACAAGCAAATTTAATCATTGTAGAAAAACGTTAAACAACTTTTTAAACATTTTAACAAAATGGACCAAAGTGTACTGGGAGCTTTTTCTTCAATATCTGGACAGCAGGAAACCGGTTTTGGGAACATGCCGTCAAAAACAACGTTTATAAATTACCCAAACATCGGAAATATTTTTATTGAAACAGAATCGAATTTTTTAGAAAGAAATAACCAGAGTTTTGATACGATTAAACATTCTACCATGAAGTCAAAGCGACCACAAAATAAAAACAAGAAAATGACTAATCGGCAATTTGGTGATGATATTACAAATTTTGGGCCTTTTCAAAGACAGTTACCAAGAGTTGAGACTTGCAACCATGTTCCTTTAACAAATGAAAACTTTTCTCCAACATACAATTATAAATCACTGAGAAATGACAACGACGAAACAATGTTTCCAAACTCAAAAAGAGCGAGAACTGAAATCCACGATCCATCGGCGATAAGATCGTCAAGATCAAATAAGGCTATGTTTTCGAGACGTAAAATTGCGAGCGAAAGAGAGAAGCTCCGAATGCAGAAGATATCCAATCAATTCCAAGTATTGAAAAGTGTCCTGCCGCCTACCAGATTCGCAGGAAACGGAAGGGTAACGAAAATCGAAATTCTCCACCAAGCGA

TGGATTA

Moocci.Ets.b probe (VERIFIED SEQUENCE):

ATAGCTTTGATAGTGGCAGTGATTGCCAACAAGAACTTGGATGTCATATACCAACACCCCTACCTCCAGTATCTAATCCTTCAGTGTTCAGAAGAAGTTGTTCTCAAGATGAAAGTGGAGGCAAGGTTCAGTTACACACAATGACTCCACTAAGGGAAGAAGAGTCCTCAATGTACCCACACCTTGAACACCTTCACAGATCCCAGTCAAATGTTCCATCCAGTTTGCCATGTGTCAGTAACCAAAGTCGAAGGCCATCTGACATCTCTCACCCATTTCCAACCCCAACCACCCCTAATCTACCTCCATTTCCCCAACCCCAAGATGGAAAATGTGTTACACCATCTGACCCCAGACACCCTCCAGGACCCCAAGATAACTGGCAAGATCTGTATTACGCTAACCTTGCTAATTACCATCACCACCAACATATGGAAAACATCAAGCGTCAGGAAATAGCTCGAGGTAAACAATGTAACAGTAGTATCCTGTCATATAAACGTTGTGTTGACGTCACAAACTACTTCCTTTATGGAATTATATGTGACTTGTGTATTGTTTATATAGTTTATATTAACGTTAATTGCTAAAGTGAATCATTGGTTTATTTTCTCTATTTAAAAGTCTCTTGATTTTTAGATAAGCTTCATGGTAATATTCATGGAAATCATCCTGTACCTCACCCTGCTTATCCAAATGTTAAGCATGAAAATGTTCCACACCAGGGTTACTGGGGTCCCCAGCAGCACCCACAGTTCCACCAACAGACAATGAAACCCTATGGTATGGAAGAGCGTGCCATGAAGGACCGGCAGTACTTCGGGCCACCAGGAATGTTCCCACCTGGTGGAGAATTTCCAAGCAATCCTGTAATACCTGGAG

Moocci.FoxF probe:

AATTTACACTGCAGTTCGTCGCTTGCGATCATGAGTCCAACCACACATGAGATGCGAGGATCTCTCAGTCAGCCTCTTCACCACTCGATACCTTCTGTTCAGAATCAGTCAGCACCTTCGTTACAAAACCAGCAGCATATTAACCCTTTACAACACCAGGATCAAATACCAGGACAAGGAGGACCAATGGATGCGTACAGAGGAAGAGCGTTATCAGATAATACTCACCAGGGTCTCCACATCAGCCATTCTCATGATGCTCTTGGCCTTCAGCAGCAGATGCAATATCAGCACCAACACAACGCTTTGTACAGATCCCAATCTATGATTCCGCAACCTGGAGTTCATCTACCTCCGAGCGTCGATAGCGTGGTGGCTCCACCTCGGCCAGGAAGCAACGAAATTCCGCACAATATCTCAATGGAACGAAAAACCTCACAAAGTTCTCCGATCTCCGAGAGTCGCCACCCTTTGTCCGACACCAGCTTGGATGACAGCAACGCGAACAATAATAGTAACAACAGAACACTGTCAGAAGATCCAGAAATTTCTGCAGATTTGAACAATACGGCTGCCTCAATGACGACAGGATCAGAAGCTTCAACATCCACTGAAGAAGATGGAAGCGATGGAGGAGTCGCAGTTAATAAAAAGAGTGGTGGTTCATCTAACGCCGCGGGACACAGACGACCAGAAAAACCCCCTTATTCGTATATCGCACTGATTGTAATGGCGATTCAAAGTTCACAAACCAAAAAATTAACGTTGAGTGAAATTTACAACTTCTTGCAAAGCAAATTTGAATTTTTCCGAGGTTCTTATCAAGGATGGAAAAACTCTGTAAGACATAACCTATCATTAAATGAATGTTTTATCAAATTGCCAAAAGGTCTTGGCCGTCCGGGAAAAGGACATTACTGGACCATCGATCCCGCTAGCGAATTCATGTTCGAAGAAGGATCTTTTCGAAGACGACCACGGGGATTTCGACGAAAGTGTCAAGCTCTTAAACCTTATGGCATTTTCGGTGGACCTCCACCAGGTTTAATGGGGCCACAAAGTTATCCACATCCTGATATGTTTCCCCAAGTAAGTGGGTTACCTCATGGTCATATGCCACCTCCACGCCACCAGCCCAATTTAATGGGATTTGACCCATCTGGAATGGGATTCTTAAATGGAGGAGCAGCTCCTATCGCTTCAAACAGAAGTCCAACGTCGCCACTTACTCCAAAGCCACCGGGAACCCCACATTCGCCAAGTAATCACACTGGGAGATTATACGGATCACCAAGTGCCGTAACCGGGCCGAGTCCCAACGATAGCAGGGCGTCCCAAATTTTGCATTCCCCAGCTAGCATGACAACAGCCAATCCACACTATCCTTCGGCCATGTTTAGTTGGCCAAGTCCAGGACCTCATCCCGGAACATACATTCGCCACGACGCAACTTCAACGGCCGCATCTATTACAGAAACACAAAATCATTTCATGAACGCTGCAGGTTCGCGAATCGAACACCATCCATTTTACGCTTCCGCTGACCGAGCTAACATCGCTTAT

Moocci.Hand-r probe (VERIFIED SEQUENCE):

ATTATAAAACTTAAGCATCAAAGTAATAAAATTAAAAAATGTTCACCGAAAATTTCTCGGACTACCTCCAAAACATTCCAAGCTCACGGTATTCAGTTGCCAGGCAACACGCCAACTTGAATGACGTTAATAGTGGATTCAACCAAGCGCAAAACTACGGAGATGAGAATTTTAGCTTCGGCACGAACAAAAGTTTTCAAAAACCTCAGGTGACAAACCGGTGTTCTGATTGGAGTAGCACCCGACCACGTGTTTTGCAAGATATTACATCACAGCTAAACGAATGCGGCGCACCTTTCGGGGCAACTTTTAACAACGTTCAGACTAGCTATACCGAGAACTTCAACGAATGTAGCGGGTTCGAGCCATGGCCAAAGCAAGTCCACCAACACCAAATCTTTCCCGAACAAAATTTGCAACAAATTGAACCACAATGGAAACAGCAAAGCTCTTGGGCGCCATCTCGGGAGCAGTCGACGATGTACCAGAGTCACAGGATGCGCACGAACAGGCGTAAGCGCGAACGGAACATGAACATCAACCACGCGTTCGAGTCTCTGCGGTGCCGCATCCCAAACCTGCCGACCGACACGAAGGTTTCAAAAATCCGCATCCTCCAGTTGGCGTGCGAGTACA

Moocci.Gata4/5/6 5’RACE (VERIFIED SEQUENCE):

ATTGGAGTATTTGGTTGTATTAAGACATATAGTTTTTAATTTTGATTTTATTTAAGTTGGTATTAAATTTTGTTGAAAATGTATGTGCAAGAAAAAAATGGGGGACAAAACGATAAACTAGCTCATGTGTACTTCCCCCCATCTACACAACTCTACACCAGCTCAAGGAAGCAAAATCCTGATACGAAGGCTTATCTCATAGAACCGGCGGATTTTAATCAAAATGCGGGAGATATCCACATCACCCCACCAGCTCAGAGCAGACAAATTAAACATGAATCTGAATCTTCATGGAATGACTCATCTGCCAGCACAACGTCAAACAACAATACGCCCTCTCGTGGCTCTCCCAACGTGAGCAACAGAAGCGCTACGCAAAATCCCAGGTCCAATAACAGCCCAATTGAAGACTCCGGAGTGGAAGAGGATCGTGGCTCGGATAACGCAACAACAGATACCAGTGGCCGCCACAGTCTAGGAAGTAGCAATGGAAGTCTGACAGAAAACACAAGTTATGATTCTGCAAATCAACAACAAATTCTACCTTACGCGAGTGCACGGTATGTTACGCAATACTCCACTCAGCCATTTCCTCATGATTACCAGAATAAAACTGGAAGTATTTCACCTGGATTGGATTTGTCAGGAAGCTATGCAAACGAGGGGGTAAATTATTCGTCCTCAATCAACTATAATTCATACCCTACAAACCCCACATTTGTAGCGGGGTCAAATTATGGATCATCGTACCCTTTGCCTTCATATGCCCCCAGCAGCCACCAAACCAATCCGGCTTTACACCACACCACTGGTTTGGCTCTAAGTGGATCCACAACTCAACATGCAAGTACTGCGACTGGACTTCCCCAAGGTTACCCATACCCTTCCACACAAGAAATGTGGGGTCTTGGAGCCACAGGCGGAGCCTATGACGCCACAACAGCAATGGCGTATCAAATGCAACTAAGACCGACTGCTCTATCTCAATTTGGGGGATACCAAGAGCAACGTGAATGTGTGAATTGTGGAGCCGCGTCAACTCGAGTTTGGCGACCAGATTCCTTGGGTCATTTCCTGTGCGACACGTGCGCCATTGCAGCAGGTCAACATGGACGTTCCTCCTCTTCACTGAAGGCCAAAGGAAAACTGAACTCCTGTCGTAATCAAATTTGCTCAAATTGTTCAACGACTGTGACAACTTTATGGCGAAGAAGCCCAGAAGGAAACTCG

Moocci.Nk4 3’RACE (VERIFIED SEQUENCE):

TCGACCGCGTACAAGACGAAAGCCAAGGGTTCTTTTCTCACAAGCGCAAGTTTTTGAACTTGAACGACGATTTAAACAACAACGTTATCTGTCAGCCCCAGAACGCGAACATTTGGCTCAGATGTTAAAGTTGACTTCAACCCAGGTTAAAATTTGGTTTCAAAACCGGCGATACAAATGCAAACGACAACGTCAAGATAAAAGCTTAGAACTTGTAGGCGCCTGTCCACCACGACGTGTTGCTGTTCCAGTGCTAGTAAGAGATGGAAAACCCTGTTTGGGAGGACCAATGGCAGGTGGTAACCCTTCCCTACAAGCTCCATACTCTGCTCCTTATAATGTCACTGTCACTCCATACCATAGTTATCCAAGTTCATATAATTCTTGTGGGTACAATTCTTATCCCCAGCCAAACCCTCACGCCAATTACGCAAGTAGCGCAGCTACATATGCCCCAACAGCAGGTTACAACCTTGGTGGACCTTCAATTGCTTCCAATCCATCAGTCGGGAGCGTTGGTGGAATTCCAGCTGGACCTATACATCAATCTGGCCAAATGACTATGGGACCATCCCATTTCGCACCCTCCATGACAAGTCAAGTACCTCAACGCAACCCGCATGGTACAAGCGACTATATGTATAAGCTTGGATTATGTACATAAAATTAAAGCTTTACTTTAATACAATGACGTGCTGCAAGGATTTTCGTGCAAAACGTAGATTTTTCACGCAGTTTTGCGAATAATACGGGCTGCTGAAATTTGGAAAGTGCAATCTATACGTGCCTTATCAGTGATATTAACGCAATCATGCAGAAAATTCGCAGCTAAATTCCCCATGACAATACTTGAAACAAATATTTTGTTAATTATTTTTACATTCATTTATTGATATATTTAAGTATAACAGTGTAATAATGAATGTATGACTAAACTGCTTGCAATTTTTACAACTTTACTTTCCATTTTTTACTCGTATGGTGATTATTTTTTAAATAAATGTGTTGCTTTAATTTTTAAAAAAAAAAAAAAA

Moocci.RhoD/F 5’RACE (VERIFIED SEQUENCE):

ATTGGAGTATTTGGTTGTATTAAGGTTTAAAATAAGCTGTATTAAAATAAAAATGTAGAAACTAAAAGCCTTTAGTTACGTATGTTTTCTTGAGGGAAGAATCGGGAATAAAACGTATTGGAAACTTTTATATTGAATTTATTTATGACGAAAACAAATCGATGAAACACGATGGAGAGGAAAAATAACAACAATAGAAAATTTTCTGAGCATATTTATATCAGCAGCGTTGATTCAAAAACCTGCCGAGCTAAGCCGCACAACACCGATAAAGACGGCGATTTACCGCGGATTAAAATCGTTGTCGTAGGCGACGGGGGAAGCGGGAAAACTTCACTTTTTATTGTATTCACCCAGCGAGAGTTTCCGGAGGTATATATACCCACTGTATTCGAAAACCACACTGCCGAAGTTGAATTTCACACAAAGCGACTTTTTCTACTTCTATGGGACACAGCGGGCCAAGAAGATTATGATCGGTTACGGCCTTTATCTTATGACGATTCTGACGTCATACTCATATGTTATGACGTCATGAATCCATCCAGTTTTGAAAATGTAAAGGTTCGCTGGGAGCCTGAAATTCGGCAC

Moocci.Tbx1/10 5’RACE (VERIFIED SEQUENCE):

ATTGGAGTATTTGGTTGTATTAAGTTCACTTTTCATTTAATTAAGTATTTTATAACATCGAAGATTTACCGGTAAGGACGCTGAGTTAAAAGCGTTAAAAAACGGCGATCTGTCGTGATTTTTTCGGGTGTTTAATGCTAAAGTATTCTAGCTAACATTTAGTGAATCTGAAAAAGATATATAACAACATCTTCTGCAATAGAAATATTCCCCGGACAGTCTGTTAAACAAGACAATCCAACGGACAGTTTCAAAGGTATTCCAGGCATTGACTTTACCCAAATTAGAGCTACAAAAGGGTTAAACCAGATTTTAATTTATTACCCCAACCCCTTAAATGACAAGCGGGATTGGAATGATGGGGCCTAGTGGGGGCCATTACCCGCCCCATTACGAGTACACGCTTTCTCATCAGGATTATGGAACGGAGTGTCAACAATATAACGAATATTCTATGCAGTACCAACGTGCGGAATTCCAACACCGTACGCCATCTCGTGATGAGAATTCCCAGCCAATGAGTGAGAAAAACCACAATAACTTTTCACCCAAGAATCATTCAAGTTTAAGAGTGGAAAATTTTCATGAACGGGAAGAGAAACCGTCACAAAGCGACATACAAAACGTTGGTTACAACAACAACACTTACGATGATAAAATAAGCTCCATGGCGCAAGCGCGACAATTATTAAACATGCAGACCGCTGAGCACGCCACTAGTGGACACATCGACAATAAGTACGCACGGAGTAGCTGTTCGATGAGCGATGACGTCAATAATGTGGCGTTGTTGGAAGCGAGTGTGTCCAAACCCCTGTCGCCATTCAAAGCGGACGCGCTACAAAATCACAACCACAACAACAACAGTAACATGAGTCGTATCTCGCCGAACGAAAACGACTCCACCATGTCCCCTAAAATGGAAGGGTCTCCAAGTATGGTGTCATCACGAACTGATATGACGTCAATGAATGATGAAATGCGTGATGACGTCATGGACACGCAAGGGAAAGAGGCGGGCAAAAAGAATCACAAGGTGTCGTCAGTGTACGCTCGATTGGAAATGAAGAATCTTTGGGATGAATTCAACGAACTTGGAACCGAAATGATAGTCACTAAAGCCGGAAGACGGATGTTCCCAACATTTCAAGTCAAGTTGTTCGGGATGGACCCAAACGCTGATTACATTCTCATGATGTCGTTTGCAACGGTGGACGACAAGCGGTACCGATATGCATTTCATAGTTCCAATTGGTTGGTAGCGGGGAAGGCGGATCCTTCAATGCCTCCACGAATCCACGTACACCCTGACTCCCCCGCCAAAGGATCTCATTGGATGAAACAGATCGTTTCCTTTGACAAACTTAAACTTACCAATAATCTACTGGATGACAATGGACACATCATCTTGAATTCGATGCACCG

Moocci.Ebf probe:

GCCGGTTTGCATAGAGCTCATTTTGAAAAGCAGCCGCCAAGCAATCTTCGCAAAAGCAACTTCTTTCATTTTGTTTTGGCTTTGTACGATAGGCAAGGTCAACCCGTGGAAGTCGAAAGAACAGCTTTTGTCGATTTCGTTGAAGGGGAACGAGAAGTTGTTACAACTAGTGGCGAGAAAACAAACAATGGAATTCACTACCGTTTGCAGTTACTCTACCACAATGGAATGCGAACGGAACAAGATTTATTCGTTCGGCTTATTGATTCGGTCACAAAACAAGCAATTTCCTACGAAGGACAAGACAAAAATCCTGAAATGAGGAGAGTATTGCTAACGCATGAAATAATGTGCAGCCGGTGTTGTGACAAGAAAAGTTGTGGAAATAGAAATGAAACACCTTCCGACCCTGTTGTGATCGACAGATATTTCCTGAAATTTTTTTTAAAGTGCAATCAAAATTGTTTAAAGAACGCTGGAAATCCCAGAGACATGCGACGGTTTCAGGTTGTTATCTCAACCACAGTTCACGTGGATGGACACGTTCTTGCTGTGTCCGACAACATGTTCGTCCACAACAACTCTAAACATGGGAGAAGAGCGAGAAGGATGGAACCTTCTGACGCAACACCCGTTATTAAAGCATTAAGCCCAAGTGAAGGTTGGACTACAGGCGGCGCAACTGTTATTATAGTGGGCGACAACTTTTTTGATGGATTGCAAGTTGTCTTTGGTTCTATGATAGTTTGGAGCGAGCTGGTGACACAACATGCAATAAGAGTGCAGACACCGCCAAGACATGTGCCTGGTGTAGTAGAAGTAACATTGTCGTATAAGAACAAACAATTTTGCAAAGGTTCTCCAGGAAGATTTGTGTACACAGCCCTGAATGAACCTACTATTGATTACGGGTTTCAGAGACTGTTAAAAGCTATTCCAAGACATCCCGGCGACCCTGAACGTTTACCAAAGGAAATCGTACTAAAGCGAGCGGCTGATGTTATGGAAGCTGTGATGACAAGGTCTTATAACCAAGTCCCGGCGCCTCCTCCAGCTCCTATCCACAACGCATTTAACGGTTCGTCACCATCCATGATGACAGGAAATGTTAATGGTTACAACCACCACATGCCGTCTGCACAATATGGATTAGCAACACCTGATCGACTGGATTCCGCCAACGGTAGCGACTCCGGTAAAATATTTTTAGGAAACAAATTGGGCGGTAAAAATCTCAATTTATATATTTTAAGTATACTTTTATTTTCCAACTAAATCGAAGTATAGTTATAAAATTGTAGCTATTTATCTTTTTTTTATTATCTAGGTTATTCAAGAGGGAGTGCTTCTCCAAAAACTGGTTATTCGCCACAAGGAACACCCCATAGTACTGCCAATGTTGGATTATCTACTGTTGGGGGTAATGTCCCGCCTTATGGCAATGCCATGAATGGGTATTCATGCAATCCCACTTTCACCAATATGACAAATTCAACATCCAACATGTTTACTGGTGGTGGATTGTTTCCAACTTCTCCAAACGCATGTATGAATGCATTGCCAACATGCGGAAGCACACCAGGTATATTTTCATTTTCTCCAGCAAACATGATATCAGCTGTAAAGCAAAAAAGTGCTTTTGCGCCGGTAGTTCGTACTCACAACTCGCCGTCTCCAA

Moocci.Dlx.a 3’RACE (VERIFIED SEQUENCE):

GCCGGAAACCCCGCACCATCTACACCAGCTTCCAATTGCAACAGTTAATACGACGGTTCCAACGCACGCAATACCTTGCGTTGCCGGAACGCGCAGAACTCGCTGCTTCGCTCGGAGTTACTCAAACACAGGTTAAAATTTGGTTCCAGAATCGAAGATCGAAATGTAAGAAAATTTTAAAACAGCAGATTCTTCACAACAAACAATCTGGAACATCAGGAGGAAACGACTTCTTTGTTTCTCATCCCAACTCCTCCAGGATGTCTTTTCCGGGTGTAATTGGATCGCAAATGGGAAGCGAGTTAGCGGCGAACCAAACGACCGCGGATCAAAACCCATACCGTTACAACCAACATAACAGCCCTCAGCAATGGGGTGTTCATGGGTTGCCACATCACTCGCATTTTTCAAACCGTGAACAATTTTCATCTTCTATAGGACAAAACAGCTTAAACACCAACAGAAGTAATCTCGACTCATGGTCAATGGTAATACCGAATTCAACAAAACGGTCGTGGAGTTATTCTGACTTTCCTCAAAGTCCAACTGCAAATCCACAAATAGGTGCTTCATATACCCACCGCAATTTTCTATCGACCACCAATGATATAGGCAGACACCCGCAAATGGATTCGAACCCATACTCTGTACCGTCGTTGGATCACAACTCAGCAATGTTTCAAAATAATCCAACGTTACCTTGGAGTTAAAATAACAAGGATTTATTTTTAACCGTAAACAATTACATACAATTAAATAAACTTTATTGTTTACTTTTATGTCTTATATCTTCACGCATGGTTTAATATTAGTATGCCGGTTCTTATGTTATATGATTTTTTTAATAAAAAAAAAAAAAAA

Moocci.Tbx6-r.a probe (VERIFIED SEQUENCE):

GTCGGAACAGAAATGATTGTAACAAAAACTGGACGACGAATGTTTCCAGGCTACAGAATAAAAATCACAGGTCTTGACCCGGGTGCAAAATATTGCGTTATGATGGACATCGTAAACGTAGATGACAATCGTTATAAATTTCAGCATGGTGAATGGACAGTTGCTGGAAGAGGAGAACCTCATCTTCCCCAAAGATATTTTCTTCATCCAGATTCACCTGCAACTGGAAATAAATGGATGAAAGAACCTATTTCATTTCATAAAGTAAAACTAACAAACTCTGTAGGACAAGACATAGATGGAAAGGTTGTTTTGAATTCGATGCACCGGTATCAGCCGCGTGTTCATGTGGTCCAATCAGATGACCCATATTCTGTTCACACTCAACCCATGTACACTTTTGCTTTCCCACAAACAGTTTTTATAACCGTAACCGCATACCAAAACGCAGAGGTA

Moocci.Tbx6-r.b 5’RACE (VERIFIED SEQUENCE):

ATCATTTACGTTTCGTTTATATCTTAAATTATTTTAATATACTAACAAATATGGAGAATTTAATTTCGATCAACGCTGAGAAATTTAAAAATTTACCGGTACAAGATGTTTACAACAAAGCAGAATGCACGGCTTTACAAGGAGATATAATGAAATACCAGAAAAATTTTAATTCGGTGCAGAATTACAACAGTCCAATCCAAAATGATCAATGGAACACCGATTATCAACACAATTTACCAGAAGCTACGTACGGAAATCAAACTGAAATCCTAAACAGCTACCACGTGGGTGGGATCAACGTGACTTTATGTCAAGCTGACCTATGGCAACAGTTTCATAGAGAAGGAACAGAAATGATTTTGACTAAAAATGGAAGAAGGATTTTTCCTGGATTTCGCATAAAATTAAGCGGACTAAAACCCGACGGATACTATTGCGTTTATCTCGATGCAATTAGTTTAGATAATCATAGATACAAATTTCAAGATGGTGAATGGATGATAGCTGGAAAAAGTGAACCACAACCACCACGCAGATTGGCTTTTCATCCAGATTCTGCATCTCTTGGTAGCGCATGGATGAAAAATACAGTTTCGTTTCATAAAGTTAAGCTGAGCAATTCTGTTTCTTGTCGCGATAAAAATGCTTTAATTTTAAATTCTATGCATCGTTATCGACTACGAGTTCATGTAGTAGAATGCTCTGATATTTCCATGGCGCATTTGTTACCTTCGCACTGTACAGTTTTCAATGAAACAAGTTTTGTGACAGTTACTGCTTATCAAAATCATGAAGTTACAAAACTTAAAATTGCAAACAATCCCTATGCTAAGGGTTTTCGAAAAGATGGCGCTAGAGGTTCAAAGTCTCTTTCAAGCTCGGATAACGAATATGAGAGTTCTAGCAAACGACCAAATATCTCCTCCTTTACAGGCGGTCATCAGCA

Moocci.Lhx3/4.a 5’RACE (VERIFIED SEQUENCE):

ATTTGGTTGTATTAAGAAATATTAAAACTATTTGTTTGAACTTTGTAAAGTTTGTTTAATCGATATCGAAAACAATGGAGACAGTTTCTTCGAAATGCCTCGGCGAAAACTTTTACACTCGATATGATGATACAAACAAAATGGCGGACAGTGAAATAGACATATCTCGCGAAAATTCTAATCACATGTTTAATGACGTCACAAGTGCGATGTTTGCAGAGGAATGTGACGACACTGAGGTTTATGAAGATGACATCTTGTTTGATAATGATGAAGTCATTAAAACAAACTTATCGTCGTTTGTTGGAAATCACAAGAAAGATTATTTTTCAACTTCCACGCCGCAAAGAGGTACAAAAACAAAACAATCTTCCGGGTTAACAAGCGGGTTACCAGCAAAAATGAATCCTTCGTTAGATAAAAATGTGAACAACAAAAATGTTTTGTACTCCCTTCTAGAAAATGGAAAAAAAATTTCAAATAACTTTTCAAAGTGCACTGGTTGTGGACACCACATATTAGACAGGTTCATACTGAAGGTACAGGATAAACCGTGGCACGCAAAGTGTTTAAAGTGCCACGAGTGTTTGTGCCAGTTATCGGAGAAGTGCTACTCCCGAGATAACTATGTTTTTTGCAAAGAAGACTTTTTTAAAAGATTTGGTACGAAATGTGCTGCGTGTGGACAGGGCGTTCCACCTACGGAAGTTGTGCGAAGAGCTCAAGAAAACGTTTATCATCTCGACTGTTTTTGTTGTTTTATTTGCAACGAGAAAATGGACACGGGAGATCATTTCTATTTAATCGAAGACGGAAGACTTGTTTGTAAAAACGATTACGAGCAAGTCAAAGCGAGAGATATTGATTTTGAAAGTGGATCCAAACGACCAAGGACAACAATATCAGCCAAACAGTTGGAAACTTTGAAGTCAGCCTATAACCAAAGTTCGAAACCGGCGCGACATGTGCGCGAACAACTGAGCGTGGATACAGGGCTTGATATGAGGGTGGTCCAGGTTTGGTTTCAAAATCGACGAGCAAAAGAAA

Moocci.Lhx3/4.b 3’RACE (VERIFIED SEQUENCE):

TAAAAGTTTATAAATCCATTTTTGAAAAAAAAACAATAACTATGGAATCTTTTATGTTTGAAAGTCCGACAAAGTTCAATTATTTAGTAACAAATGACTCAGGTTACAGCTCTATCCAAAGAACTCCAGACATGTTTACCCAATACTGTGATAATTTCGTAGAATTGGAAGAAATATGTCCCATACCGTTTCCTACACTGAATCAGGATGATGCAGAAGAACCAATACCAGTTAAAGAAACGCAAAGTATTTCGAATATATCCAAGTTGCTTGCAAAGATACCAAAATGTGCAGGATGCGACCTTCATATATTTGATAGTGAATTAATTCAACTCCCAAACCAATCTTGGCATGAAAAATGCATTCAATGTTCCGAATGTTCTTGTCCGTTGGATGAAAATTGTTTTGTTCATGACAATCTTCTTTTCTGCCGCAACCACTTTTACATGAAATTTGGAGCTGTATGTGCTGCATGCGGCGAAAGAATGTCTCCAAAAGAAATAGTTCGTAGAGCAGAAGAGAAAGTTTATCATGTACAGTGCTTTAAATGTACTCTATGCCAAAAACAAATGGGTAACGGAGATTGTTTTTACATGACTAAAGATTGTAGAATAATTTGTAGCCAAGATCACAGTAATTTACAATCTGAAGATTTAGCCGAAATGGAGATTAAACGCCCAAGATCCATAATAACAAACAAACAATTGGACGTTCTCAACTCAATTTACAATCAGAACGCAAAACCACCTCGTCACATCAGGGAGCAACTGAGTCGATCGACTGGTCTCG

Moocci.Aldh1a probe

CTAACAATGGTGAAACAATGGACATTATCAATCCTTCAAAAGAAACAATTATTACCAGTGTGTATTCTGCAACGAAGGAAGACGTCGGTAAAGCTGTCGATGCCGCAACTGCCGCTTTCTCGCTTGATGCACCGTGGCGGAAGATGGATGCGTCCGAACGCGGCAATTTGTTGCTGAAATTGGCGGACGCAATTGACGCAAATAAACATTATTTGGCGTGTTTGGAGAGCTTGAACGCCGGTAAACCTTACACACATTCATTCTTCATGGATACTCTCGGAATATCGAAAATACTGCGCTACTACGCCGGTTGGACGGATAAAATGCACGGAAAAACTTTTCCGGTAGATGGCGACTACCTCAGCTATACCAAAGTCAACCCAGTCGGAGTTTGTGGACTAATTCTACCGTGGAATATGCCCTTGATATCGCTAGCAATGAAATTGGCACCAGCATTGTGTTGTGGCAACACTGTCATTGTTAAACCTTCCGAAAACACGCCACTAACGGCGCTCTATGTCGCCTCATTAGCCATGGAAGTCGGGTTTCCTGCTGGTGTGATCAACGTGTTACCTGGTGGGCCATCTAGCGGTGCAGCTTTGGCTGAGCACCGAGGCTTGGCGAAAATTTCTTTAACTGGTTCAATAAAAACCGGGCAGATCGTGAGCAAAGCTGCTTCTGACAACTTTAAAAGACTGACTTTGGAGCTGTCTGGAAAGTGCGCTAATATAATATTTGCTGATACCAACGTGGACAGCGCAGTAGAGCAAGCTCATCAAGCGTCATTCATGAACCAAGGTCAAATGTGTACGTCCGGAGCTCGTACTTTCGTTCACGAGGACATCTACGAAGAATTCGTCACCAAAAGTGTTGCCCGTGCACAAAGGCGAATGGTTACAGATCCTTTCCAACTGTTTTGTGAACAGGGACCTCAGGTTAC

*M. occulta probe templates were cloned from genomic DNA, as cDNA was lacking at the time of probe preparation. Thus, some of the following sequences will contain introns in addition to exons.*

Mooccu.Mesp probe:

CCGATGTATTTACTGACCGAGTTCCAAACAAACAAACCTTTATTGCGTATCCTGGGTCTACTGCTTCACATTTCCCTGCTACAAATATCAGCTGTCAATCAAATGGTTTTGTAATCAAAAGAAACCCACTGCGTGACGTCACAAATGCGAATCACTGTTATAATTATGGAACGACAGGCAAACGAAAATTGGATCTTGAGAATTCATCGCCAAATAAAAGATCAAAGTTTAACAGCTCAAAGACGAAGAAACCAAAAGTAAAATCCCGTCGTGAAATTGCAAGTGACCGTGAA

Mooccu.FoxF probe:

ATTTCTTGCAAAGTAAATTCGAATTCTTTCGTGGGTCATATCAAGGATGGAAGAATTCAGTTCGACACAATCTTTCATTGAACGAATGTTTCATCAAACTTCCTAAAGGTCTTGGACGTCCAGGCAAAGGACACTACTGGACAATTGATCCAGCAAGTGAATTCATGTTTGAAGAAGGTTCATTTAGACGTCGTCCGCGTGGCTTCAGACGTAAATGTCAAGCGTTGAAGCCGTACGGGATATTCGGTGGACCTCCACCTGGTTTGATGGGTCCACAAAGTTACCCACACCCTGATATGTTCCCACAGGTCGGTGGAATACCACACGGACATATGCCACCGCCCAGACACCAGCACAATCTTATGGGATTTGACCCATCCCAGATGGGATTCCTGAACGGAGGATCGGTCCCAGCCAACAGGAGTCCAACTTCACCCTTGACACCAAAACCACCCAGCACGCCTCATTCGCCAAGTGCGCAGGCCGTTCGCGCATATGGATCACCGAACTCAGGAAACAGCGGACATCATTTGGAAAGCCGACCATCACAGGTGCTACATTCACCGGCAGGTATGCCGACACCAAACCCTCACTACCCGACAGCTGCCATGTTCAATTGGCCAACACCAGCCGGTCCGCATGCAGGAGCATACATTCGTCATGACGTCACAAGCACCGCTTCCTCACTGGCGGACGCACAGAGTGCTCATTTGATTAATGGAGCACCGAGGATGGACCATCATGGTTTCTACGCACCATCAGAGCGAGGGAACCATGTATCATACGATGGTAAGTTGAATATTGTGTTTATTCCAAACAAAATGGCGGGCGGATACGTTCGAATTTTACAAGCGCATTTTTGGTAGCGTCTCATTAAAGCGCTAATCTGCAAAGGAATAAGAAACAAACTCTAATTGAAACAATGGATTAATTCCTGTAATTTGCAGTTTCAAATTTCGTAATTACGGGATATAAAAATTATTATGACATAACAATGGTTTTATTGACATTTGGCAGATAATTGCGTTTATTATCGTTTCATAATCGCACAATCTGCTAATTTTCGAAATGGGCAATTTTTTTATCGGCAAATTTGCGAGCTAAATCGCTATCGGGTTACCAAATCGTAGCTGTCAAAAATTGTCACAAGAAATCTTCTAAACCGAAATTGTCCATTCGGGAATTAGAAATGCTACTGGTCAAGATAATTGTTACATCACATTGGTCATTGTGACAGAGGACGATTATTAAACAATTAAGAAATAAAAATAAACGAATTTTAATTGTTTCAATCAACTTGTCGAATTATTCACAATTAGTGCGTCTAAACAAACAAAGGTAAAATAATTATAATTTCTATGATGTATTTCTATACTAATAACTATCATTTTGACGCAATAATCACGCAATTGTCCCTTGGAACGCCTCCCCAAACCTCACGATTACGATAAATTCTACTATTTACGACTGAATAAATTCGCATTGGGTGGTAAAGTTGAAAAATTTCCGGACCAGTATTATCAATTTTTAAGGCATTTATGTAATCTGTGTATAATTGCTTGCGTTGTATCTTGCCTCGGGTCATACGAAGTACAATAAAACGATATTTAAAAAAGGAAAAATCTGATAAATTTCGATCAACAGATAACAGAAAAATGTACAACTGCGAAGCAGTCTTAATTACCAAAGCGTCGAAGTTCATCTCATATTGTTGACGAACAGCATTTCAATGTTTAACGATATACGAGATTGCCGTACATTAACGGCAGGTTATTGAACAATTTCAACGGATTTACCGTAACAAATTTCATCATTACGGTGCGATTTTTTGTCAGTTTTTCGAATTTATTTCAAAGATTTTTAATCTTAAAATCGACTGATTAATACCCCGTTACGTCACAATACGCGGCATATATAAAACGGCAATAATTATAAAATTAAACGAGATTTGTGCAATGCAATTAAGTCGGTTATTGAAGCTTTTGATCTTACAATTGGATATTTAACCGCGACACGTGCTCACGACGAAAGTTACGACTCCAATAACCAAAAGTACGACAGATATTCATCCCGAAGGATGTGTCCTTAATTATCACGTCATAATCGACACACTTTCGCGTATGCTGCGGTGCATTGTGATGAAATAAACAAATATATTTAAAAGCAATTTGTTTTTGAACGCAAAACAAGTTTTCAATTTTACGGCGAAAAAAACTTCGCTCGTCCAAAACGCGCATGAATAAATTATCGGCTAAGGGCGCCAACAATTCGGAGTGCAAAAAAATTCAAATCGAAAATAAAATTAGTTGTTTCTTGGAGCTTATATGGATCCCCTGATGTTGCTCTGCCACCCACAATGTTCCGCTTTTGGTAAAACCCATGGAAATATGACGCCAGTGAACCGACGATATTTAAAACATTTCTTCAATTTGGTTATCCGCGCTAATCGCCGAACTTTATCTCCATTTCGTCCCAGCTTTTTGCGGAATGCGTTTTTGACGGAATCCAGATCCGCTGGAACCAGATTTAAAAAATGCAATATCAACACGTCATAATGCGAATTATAACGTAACAATTTAATCGCATTATGTCTAAATAATCACCATCAACTCTCATGGCAATCACTGAAAAATCAAAATTCACATGAGCAACTTTGTAAAATCCAAAAGCAATCTTTAAGGGGCAGTAGAGCTACCTACTAATCCACGAGGTTTTAACAGAGCTTATTACCCTGCTTTCCCGTTAGTCCCAACGTAAGATTGGTAAATTAACAGATCAAAAATCACCAAACAGATTCGTCAAACAGAATCGTAAATAGTTGTAACTGACAAAACAACCTACTTTCATAAGACCACAGGCATTATAAATCCTGACAAACAGGCATTTCACATCGCCTGTACTTTGCACATTAGATACTTGTAATTAATTGCCAGAAGAATTTCATTATAATAATACCATGTCTAAAACTACACATTGAAATTCAAAAAAATAAACCTCGTTTTTTTAACAGGAAGCGGAATGAAATTTAAAACGGAANNNNNNNNNNGCAAACAGGCATTTCACACCGCCTGTACTTTACACATTAAATACTTGTAATTAATTGCCAGAAGAATTTCATTATAATAATATCATGTCTAAAAATACACATTGAAATTCAACAAAATAAACCTCGTTTTTTTTTAACAGGAAGCGGAATGAAATTTAAAACGGAATGCGCCGTGGATTCTTATGGTGTTGGTGAAAGAAAAGGACCTTACCCTGCTCTACCACAAGGCTTACCCAACACATCAGTATACCCAACTAACTACTATGACACTAAAAGTTGCGCAGTATGAACAACTAATATCTAACAATCGTTGTGACGTCATAAGAGCAATATTATTTATG

Mooccu.Hand-r probe:

AACGCTACTGAGTGGGTTCCCAACCAACAACCTCGTGCTTTACAAGATATCACGTCACAAATGAACGCTGGTTATTCAAACATCCCATCAAAGCCATGCAACTTGCCAAGATGGAAACAATCTGTAACAGCCATTAATCAGATATCACCAGAAAGTCATATGAGGTGTGCGCCGATCCACTGGGAACATGAAAATAATATCCAAAATTGTGAAGAGTTTCCACCCCAGCAATCCACTGGTTGGATTCATGAAAAGCCACCTTGTGGAACGACAACTTTCCAAAATCATCGCTTGAGGACCAACAGGAGAAAACGTGAAAGAAACATTAACATTAACAATGCGTTTGATGTGCTTCGGAATCGAATCCCAAACTTACCACAAGACACTAAAATATCGAAGATTCGAATCTTACAATTGGCATGTGAGTACATTCGTCATCTTGGTAAAACATTGGAGGAAGATGACGAGAAGAATCAACATTGCTTCACGAATGAACATGTTCTTGAAAGTGAAGCCGAAACCAACACCGAATTCACCATCAACCCACTAACAGTTCGCGCCAAACGACGACGAATCGATTGGAGTCGGATACAGAACGAAATTGAACGACACGAAA

Mooccu.Aldh1a probe (VERIFIED SEQUENCE):

GAAGACGTGGACAAAGCTGTGCAAGCTGCAAAAGCATCCGCTCACGTCGACTCCAAGTGGAGGAAGATGGATGCCTCTGATAGAGGAAGTTTGATGATGAAGTTGGCGGATGCTATTGATGAAAACAAACATTATCTTGCTGTAAGATGATGAAAATATTGCAAATAATTCCTTTTTGACACAGATTTTTAGTGTTTGGAAAGCTTGAACAGCGGGAAACCTTACACACATACGTTTTTCATGGATACCACCGGGATATCAAAAATTATGCGGTATTATGCGGGATGGACAGATAAAATACATGGAAAAACTTTACCTGTCGATGGGGATTATTTATGCTTCACGAACGTCGTGCCATTGGGAGTTTGTGGATTGTTGTTACCAGTGAGTTGTAATAAAAACAAATCGATTGTGTCACAGTCAATTTATTGTTATTTTCAAAGTGGAACATGCCGTTAATCTCGTTGGGCATGAAGTTGGCTCCAGCACTTTGTTGTGGGAACACTGTCGTTTTAAAACCATCCGAATACACACCACTGACAACATTGTACATTGCGTCACTTGCCAAACAAGTTGGCTTTCCTGAAGGTGTTATAAACGTCGTGCCAGGAGGACCAGAGACCGGAAAAGCAATAA

Ciinte.Dlx.a probe:

ATGGCAACTGTAACCGAGACGGTGAACCAGCAGATCCCGAATCCAATTGACGACACTTCAGGCGCGTCATCAAACTCAACTACGTCACCAGGCAATGACGTCAGACCGTGGGTGCATGACGTCAAACGGGAACCCCCAGAAACTAATTGCTACCAACAGCACGCCACAAGTAGCAATATACCAGAACAGCCATATCATCACGGACTTTTTCCTATGAACAGTTTCAACGCTCAGTACAATAACAACAACCAAAAACCCGCTGTAACACCGGAAAATGGCCGAGGTTATTCATACAACCATTATAGCTACCCGGGTACTACCTTGCCAAATCATCATGTACCACAAGATACATCGCCCTCGTTTTTGGAAAGGAAAAGAATACACGAACCGGGGTTCGAAGCTGGGCACTTCGTGTCCAAAGCAACAAACAATTTCTCGCACCGAACGCCTTATTTTCACGAAACCCCGAATTACAACGTAAACCATCACCAGCCACCCATGCGAGGAGTTTCAAGTCCTCGGAGTGTGGACTCCCAGGAGCAGGTGTTTCAAAACAATCCAAGTGTGCCAGGAATTTCGGAAGCGGAGGAAAAGTTTAATCAATCATTGAGTGGAAACAGTTTCTATGGCGACCATGACGAAAACGCCAGAGACTCCGACACGGATACTCCGTCGGTACAAAATGGCGACGGCGGACGAGTGAAGGGCAAAAAGACTAGGAAACCACGCACAATATACACGAGCTACCAACTCCAGCAGCTCGTTCGAAGGTTTCAAAGAACTCAGTATCTTGCTCTACCAGAAAGAGCAGAACTAGCTGCTTCACTTGGAGTAACTCAAACCCAGATCAAAATATGGTTTCAAAACCGACGTTCGAAGTATAAGAAGCTTTTAAAGCAGCAACTTTTACATAAGCATCATGGTGCGGCTGATATCATGTCAATGGGCTTTGGGTTCGGCGGACAGCAGATCGACGAGCAATCAGGACCATACATGACGTCGCCTTACTCGGAACAATATACACCCGAGAGCCAGGGGTGGGATAATGAAATGACAAGAAGTAGGAATGACCCATATCATCAGCGTCAGCAACCGCTGGTGGATCAAAACCAGTACCCGGTGAATATTAAGGAGTCTGGAGAGTTACCTGACTGCCGTATGGCGGTCCCACAGCTGCATTGGGAAGCCCAGTACCCAATGAATCCTAACCACGCGAGTCCTTATTCGAACATACCTGCTACACCACACATGGGGATCCCACTTAACGGGAGAAACCTACCAGGTGTAGTGGATTCGCACCATACTGACCCGGGCACAGCGGGCATGCGAAGAGACGGTATTTTGCAATGGGGCAGTCCATAGAT

**CIS-REGULATORY SEQUENCES FROM *MOLGULA* SPECIES**

Coordinates are relative to base “A” of the predicted start codon (highlighted in green when included in sequence) encoded in the mRNA-like strand of the gene in question, and are imprecise due to sequencing gaps and/or polymorphisms between sequenced genome and those of individuals used for cloning of sequences. Primers used to PCR-amplify the sequences from genomic DNA are underlined. XhoI site used to stitch together *Moocci.FoxF* TVC enhancer to the basal promoter of *Moocci.FoxF* is highlighted in cyan.

For expression of secreted or transmembrane molecules such as extracellular ligands and their receptors, we modified the drivers as to not include the start codon or follow it with a stop codon.

Moocci.Mesp -1148/+21:

5’GTGTTTAGCGATCACGGTTCAAGTTTAGGCTACATCGCTTTTTCCATCGGTTTGCCTAAACTTGCATGCTGTATACAGTATTAAGAAGGTCTTGTTTAACAATGTTCCTTAAATGTTATTGACATACAAAGCAAACTTTGTGTTAAAAGCCTACTTTAATGTGAAGACCAACATAAAAAAATTATCGGATAGTATAACTTACTCAAAAAAGTTAAGTCTTTGTCCTCTTTGCAAAGCTGCAGTAACTTTATCGAATCCCCCAAGATACACGGATGTTCTGATGATAACTAGAAGCATCCCCACTATCATTACTCCAGCTTGTACAACGTCTGTCCATATCACACCTTTTAAACCACCCTGGAAATTAAATCATTATTATTATTAAAGATAAAAACCATTTATAAGCCTATTTACAATACAGTTCCAAATTTAATCTGCCAATTCGATTGAACGTTTTTCAATGAAGTAAAATATCTCTCACAATCTTGTATTGAGCCTTTAAAATAGCCTTTTAAATGAAAATCTATATCTGACAAAATAGGCTTTGACGTGATTTGAACGCCGTACCTCATGCTCGTATAAATACCTAATTTGTTGCGGAATTTCTACTTCAATATAAAAAGTATGCAAATACATACATAATAGTTATTGAAAAGAATTTAAATCATCAAATTTTATGTCTGTTGTAGCCTAAATAGTGTTTTATTTAAGTAAATCTACACTGCGCTTCAGTTCAAACCAACCGTGCCTTGAACTACGGGCAGTGCGGTTGCTATAGACGTTATGCTTACGTAAACTAGTGCAAAGAATTTGTTGGAAAGTGTTAATTGATATCCGACCACCGACTGTGTTATCTTATCGTTTTGATAGATAAGAGCTGATAACACTGATAATAGACTAATTAACTATAGCAGTTAATTTTTATTAAAATTTTCACTTTTCGGACATGAAAATTTGTCTAAAACCCAAACTTCCAAACTTCATCTATATAAGGAAAGAATTTTATTGAAATGGCATACTCAATTCATCAGTATATATCATCTATATCTATATACGGTGGGTAATACGTCGTAATTGATATCGGACAAAGTAAAAACTGCATATTGAAGCGTTAGAAGAAAGTTGTTCAATTCAACATTTTAACAAAATGGACCAAAGTGTACTGGGA3’

Moocci.FoxF -1517/+21:

5’TTGTCGTACGACCACTTAGCTTAATTACTACGTATTAACGAATAATAGTAACCAGTAAGTGCTTTAATCACAGGACACCTATTAATTAATGGTGAAGGGCGTTATATCTCATTAAGGCGGAGTTCTCTGAACTTTTAATTCCTAATATAACTTTCTATATACCTGGTTCCGTTAATCTGCGGTTTTTTAAATAATAAAAAAGTTCAAGGTTCAAAAATTCAGGTGAAGAGTTTTATGACTTGTTTTTCAGAAATGTTTGTTAATGATTTTTATGGCTAGTGACGTCACACCATCTATTTTCATACAGTAAAATTTAGCAACGTCAGCGAACGAGTAACAGGCAGTAAAACGTCGTTAATATTCTCCTACATTGGAGCAATTAGCAGATACATTGGGTCGGAAGGGTGACCACATTAAACCTAGGTTCAGTCCAGTGTCATAGTCGGTACGTTCGCGGAAGAACTAATTAACGCGAGCGGATAATAAAACTGGCGGCGCAGTGGAGCGACGAATATTTGAGTTCTGTATCAGCACAGATCCGTGCAAAAAAATGCGTAGTAAAGAAAAAGCGTCGGATTTGGTAAAGTCTCGTCATCGAGATATGTAATTTTGCGATGAGTAGTTCATATTCATTATCTTCCCGATAGGTGCGATATAGTGGTGTAGATTTAAAGAGAGATGAAATAATATTGTTTACGACGTAATGTAAACTAGTTGGAATGTTTATTTACCAACAAGAATTTACGCTGGATATATAAGATTATAGTTTACACTGTATATAAACAATAAAAAAATTGGAAACATTTTAAAGAAAAGTATAAATTTTCTAAAGAAGTTGATTTTTAGGGGAGTAATATCTCAATTTTGTTAATTTTTATTGGTCCAAATATATTTCAAATTAGACGCATTAGAAATAATTTTCGCCCACTTCGCAATATGTTAAATTAGATTTTAATTTAACCGGTAAGCGTTTTGGTCAAAACAGGGCAGGAAATTTAGGGACCTGTGTTTATTGTTGATTCGCAGTATATGACCGATATAGTATTAAAAGAATATTCCACGAATATAATTTGCAGTATCAGTCAGTTTCGACGGTAACAGTAGCTGTTGAAAACAAAATGGATTCCACTAATAGCCGGTTACATATAACAATTATATATTGAATTTAGCAAGTGTTTAATAGATTGTCAATCTAATCCAATAATTACCAGTTAAAATATTATGCCAGATTTTGAAAGGTATTTGTATACAGTTAAGCTTGTTTTATAACAGTGATATGTTTTTGTTGCAGTCTTATAAGTTCCGCTACAACAGTAATACGGAAACTATAAAAATCAAAGTAGTTAGATAAATATTCTATACGCAACTTGTAATTTTAATAATATTTATTTAATTCTTTTGTTATTTTAAAAAAAGTTCAACTATATATAAAGAGTTATAAATATTAATATCATTACTTACAATTTTTACTAAATCAACCAACAAGCTTCAGAAAGAAAAGAGTTTTAATATTTCAATGATTGAAATGGACGGAAAT3’

Moocci.FoxF -1517/-425 + basal promoter of Moocci.FoxF (“Moocci.FoxF[TVC]”, VERIFIED SEQUENCE):

5’TTGTCGTACGACCACTTAGCTTAATTACTACGTATTAACGAATAATAGTAACCAGTAAGTGCTTTAATCACAGGACACCTATTAATTAATGGTGAAGGGCGTTATATCTCATTAAGGCGGAGTTCTCTGAACTTTTAATTCCTAATATAACTTTCTATATACCTGGTTCCGTTAATCTGCGGTTTTTTAAATAATAAAAAAGTTCAAGGTTCAAAAATTCAGGTGAAGAGTTTTATGACTTGTTTTTCAGAAATGTTTGTTAATGATTTTTATGGCTAGTGACGTCACACCATCTATTTTCATACAGTAAAATTTAGCAACGTCAGCGAACGAGTAACAGGCAGTAAAACGTCGTTAATATTCTCCTACATTGGAGCAATTAGCAGATACATTGGGTCGGAAGGGTGACCACATTAAACCTAGGTTCAGTCCAGTGTCATAGTCGGTACGTTCGCGGAAGAACTAATTAACGCGAGCGGATAATAAAACTGGCGGCGCAGTGGAGCGACGAATATTTGAGTTCTGTATCAGCACAGATCCGTGCAAAAAAATGCGTAGTAAAGAAAAAGCGTCGGATTTGGTAAAGTCTCGTCATCGAGATATGTAATTTTGCGATGAGTAGTTCATATTCATTATCTTCCCGATAGGTGCGATATAGTGGTGTAGATTTAAAGAGAGATGAAATAATATTGTTTACGACGTAATGTAAACTAGTTGGAATGTTTATTTACCAACAAGAATTTACGCTGGATATATAAGATTATAGTTTACACTGTATATAAACAATAAAAAATTTGGAAACATTTTAAAGAAAAGTATAAATTTTCTAAAGAAGTTGATTTTTAGGGGAGTAATATCTCAATTTTGTTAATTTTTATTGGTCCAAATATATTTCAAATTAGACGCATTAGAAATAATTTTCGCCCACTTCGCAATATGTTAAATTAGATTTTAATTTAACCGGTAAGCGTTTTGGTCAAAACAGGGCAGGAAATTTAGGGACCTGTGTTTATTGTTGATTCGCAGTATATGACCGATATAGTATTAAAAGAATATTCCACGAATATAATTTGCAGTATCAGTCAGTTTCGACctcgagGCAGTCTTATAAGTTCCGCTACAACAGTAATACGGAAACTATAAAAATCAAAGTAGTTAGATAAATATTCTATACGCAACTTTTAATTTTAATAATATTTATTTAATTCTTTTGTTATTTTAAAAAAAGTTCAACTATATATAAAGAGTTATAAATATTAATATCATTACTTACAATTTTTACTAAATCAACCAACAAGCTTCAGAAAGAAAAGAGTTTTAATATTTCAATGATTGAAATGGACGGAAAT3’

Moocul.FoxF (VERIFIED SEQUENCE) -1337/+6:

5’GTAGAATGGCTGGTGCTTTGTGAGCGAAAACTTGCCGAATTACTCGCTGCAAAATTCGTAACGGATTAAGATCATCTTCGCCAACGGCAAGAGCTTTTGACAGGGCTGTTCTCTTGACGGATGGCGGAATAATTAATGTACCATTCGATAGGAGCTGATTAGACCTCGCTGTTGCGATGCATATGCTCAATGTTTATAGTAAAAACATTCGGAGCCAGAAAACTAATCAGCTAACTCCGCGATTTCATGTCACATGCCCAAGAAAAATTAACTCGGGCATCCGGACGACTTCAATTAGACCAGCGCGAATTAGCTCCGTTAATAGCGAGATAATTATTACGTATTAAAAAACCACAATTGGGGAATTATGCGGCTATAATTAGCAGACAAGGTGCTAAATGTCCATCGCGGTTGTTCTGATCTTAAAAAATCTTTCTTATTAATTCCTATAAAAACACATTTTGATATGACAGCTTGTGCGGTGGGTTTTACGATTTACTTCTGCAAAAGTTCATCCGGCGAAAATGTCGTAAATCTTTACCAGTTCAATTCGTAAGTAATTAAAAAATACGAGAGGTCATGAGGTGGTGGGCTAACGCGAAGTTCAGGCGGTATATAGCATGGTACGTTCGCGGAAAATCGCACACCGTTTCATAGTTTTACGACAGCCCCGTGGCGAGCCAGGCGGAACCAATTCGAAAAAATGCCGTAAAAGAAAAGTTTAAATCATCTCCGAACATCGACAAATTGAACTTCACAGACAGCCAGAAACAGTGAAATCGTCAGGAGAGCAAAAGCAAAATTCATCCGCAAATTTTATTGTTTGTTTACGAACTTTTGTAACCAAAATTGTTGTTTATTTATCGGCTGATATGGCGAaGTGGATGGATAATTTGGTCGGTGGACGATAAAATCAAAAACGCTATTAAAATTAAATAAACCAGGGACTTTACTAATTCAACATGAGTCGACCAGGAAGAGACGAGACCTGTTAAGTCTTTTTGTTAATTATCACAACGGGAAAGTAGGTCCTAACGATTAGAGAAAGGCAGAACAAAATGGCGAGAGTTTAACTACCGGCCTCATGTGCAATTGTTACTTTAGATAACGTTTGTCGATTGCCAGGTATAATTACAATCAAAATATCCAAGTAAATTGTTGCAAAACAAAAACTTTGTTTTCATTAACAAGTTTAGCGCAGTAAATCGGAAGGATTTGAAAGGTTTATATCAATAGAAAATTTACATTTTAATTAACATCGTTTTTGTTTCAGTATATAAAGTGTTACAGCGTAAATACTTGATTAGAATAACCAGCATCAATCCCAAAGTGTGAAATGGAA3’

Moocci.Tbx6-r.b -1226/-36 (VERIFIED SEQUENCE):

5’TATGCAAGAGTTGGTATCTTGCATAAAATTGTAATCTTTACAAAGATAATATTTCTCTGGTTTTTGTTGTAAATTATTAGTAAAAATGTTGCATTAAAATAAAACATTTTCGCCTTTTTATCAATTATTTTGTCAATTTAAAATAACAAAAATGTTTAGAAATTTAAAACAAATAACTATAAAATAAATACGCTATAAAATAAAGTATTTTACAATAAAACATGAAATTTTTTTTTGAATTTTGCAAATTAATAATTTACCGTCGGGTTATGCGCATAGAGCAACATTGCAATAGAATAATATTAACCAAGAAATAAATTGTTAAAAATATAAAAAAATGTTTATGCAAATTTTATCAAAAGATATTTGAAATTTGAATTAAAAAAATATATTTTGAAATATAAAAAAGTTTCTAATTGATGATTGCTGAGGCCTGTTCTTAATTGCTTTGTCGTTTAATTAGATCCTAATTACATGTGTAACAATGTGACTAATTGCTTTTGCTTGTGAATTGAGCATTTTCCATAAAATTTGTCTATTTTTAATTACTAGTTGTATGTATATTTCTAATAATTTTATTGTGTCTTTATTTTAATTACTTCCTTTTGACAAACTTTTGTTGTCAAGTTTTCACTGATAATTAATTACCAAATTACTTTTTTATTTTAAATTATATGAACATTTGTTGAGGGTGTAAACATATTGTTGTGTTTGATTAATATATAAAAAATTTTATGTAAATGGAAAAATGGGTTGAAAATAGTTAAAGATACAATTGCATTAAAAATATTATATATATATATTATACATTTGCATCTAATAATATTTCAAAATTTTAAAAGTGTCAATGCTAAATTCAAAATGTAATCAAAGCAATACCGGTACTGCATGTGTTATCACTGATAACTAGACAAGAGAAGTTGAAAATACGAGTGTGAATGTCTCAGTAGTCGAGCTACGCATTATCAGTATCCCGTCGTCGTTTCTCGAGCATTTCCAAAGCACTACCGACGTTAGGTGAAGACACTGATAAAGATAGCTGTCTTGGCCGCCGCAACTGCGAAACCCTCGCTACAAGTTTTACATCACAAAATACCATCAAATAAAAATGTTTGAAAAAGTTCAAGTCCTGCCGAGTGAAGTAGTATATAACGCTTTACAAATGTTTCAACATCATCATTTACGTTTCGT3’

Moocci.Hand-r -1226/+21(VERIFIED SEQUENCE):

5’AGGAAGTACTGTACCAACCTCATATTGGAGATTATACCCTTCAGAATTAAACAGCCTGTTCTTGAGCGCGGACTATCTGGTAGCCAGTACAATACCTTTTTACAGCATCGTAGAATTTAAGTACGGTCATAAAAACAAGAAATTGCTGGCACGGTCGATACTTGAACCCGTACTATTTGGTCTCCAAGTCAGTGTAGAAACACGATGAATCCTTACAACACTGTAGCATGCATACCGACGGGAAGTGCTACAACTGATACTTGAACCCTGAGCGCCTAGTCTCGAAATCAAAAGCTGCACATGCTTAAGCTGCTTAACAAAAGCGCTTTTGTTAAGCCTCGTTCTAATGGCACCAAAAAGTGCCTGCAACGAGATTCGGCGACGCATGAAGACATGAATTTCTTAGTTTTGTAGGTGTGTTGCGGCCTTTCGGGCTAGGCCTACAATAATTCTCCACTGACAATTATCGTGGACGACCGGGAAAAGTCCCAAGTCGCTATTATGCTTACGCAAACTTTCAAACAGGGGTTGGTTATTTGCGATGTACATGAGATCCAGGCTTTACCGGGGAATGACAGCTAAAAAATGTCTAAGAATATACTTGGTGCAAACAAGCCAGTTCAAAGGAACCATACTTAAAACTTTTAATCTTTCGTAGTGTGAACGAGATTTTATATCATATATTTAAAGTAAGCTAAAGTCAAAAACAACAAAACAACTTCACCTCAAATGGCGTAAAAAACGTAATAACCAAGAAAGTCCCAAAAATTTTGCGCAATTTTTAAGTTATTTAACAAAACCTGCTTTAATAAAAATAATAACAAACCCTAACATCTTGTGACCAGCTTGTTCAACAAATTCTACAAAATATTGACATAAAATTAAAGAAAATAAAGATTTACCAACACAATATTACATAACATAGACAGTAAACGATACAGATAATTTCCTGGGAAACAGAATGTCGTAGAATCGCACAAACAATTCCACCTGGCGAAGTTGAGCGGGCAGATGCGCGTGTTTGTTTTATTTTAAGCAAAACAATGCTGAAGAATAGAATCTTGCGCGACCAATTTCTTCATCGGTTCACCTTAATTAAGCAAGCAATTAGCACCGCAAACAAAGTCGGTTAATTAAAGCTTTCCTGCCATCAAATTTTGACACTCGTCTTAATTACTTTTTTCGTCGTCATAATTTATATATAAACCGACGTGGAAAATTTAAATCATTATAAAACTTAAGCATCAAAGTAATAAAATTAAAAAATGTTCACCGAAAATTTCTCC3’

Moocul.Hand-r -3060/-19:

5’TTTCTTAATCACGTCTCGTTCCTTGTATACATAAATTGGTGATTTAATTCCAGCATATGGATTGCGAATTTGTTTTAGTTTAAGTTCTTTGAGGTGTTCCGGTTTGAGGAGATATTCCTGCATTGCTCGTACAGCCGTAACAGTGTTATTCTCCGTGTATTTTTCATTAAAATCCCGTTGATTTTGTATATATTTCTTAACAACTTTCGCAATAGTTCCATCTTTCTTTTTTGTGTCTGTTGTTTTTTCAGAATCTTCGGTTTTTTCTTTGTCTGATGAACTTGATGACAAAGTCCGTTGATAGCAGTTTTGAGGAATAAACAATTTCTTACATTTTGTCACAGTATTTCTTGTAAAAGTTCTTTTTGTACATAAAATTGCCGACTTAGAACAAATATTCAACATTTGAATTGTTATTGAAACTTTTCCAGTCTTTCGTTTTCAGGTAATTGTTGATTAAGCTCGTCAATTAACGACAAAGTATGATCAAGATTTAAGCGAACTTTTCGCAACAAAGTTGACGTTTCTTCAATTTTCTTTGTGTATTCTGCGTGTTTTGCAAACTTCTTTTGTCGCTCAGTGTATGCTTGCATTAAATTGACAGCAAGTAAATCTAGGGTTTTAATTTTTTCTGTTAATCGACTTTGATCTTTTGTAACCAGTTCAGCACAATCTTTAAGATAATTTTGCAATATCAAACAAACTTTTAAAACTGCTTCTTTATCCAAACCAGAAACCAGGTCTCCTTCTTCAATCTTCCCACCTTCTTGCAAAATTGCAGACTTTAAGATTGGTAGAAAACTTGGGCAGTAAGACAACCTTTCAACGTGTTGATTTTCGTCTTGATTTAATCCACCTTCCGCAGTTCCTTGTGCAACGACTACTATTCCACTGGGTATATTTTGCTGTGAATGATTTGAATTGGTCTTTGCCGTGCTATGACTTTGTTCATTTCCCATTTTAAGTTAAAACATTGCACCAAATCTCAAAAGGCTGAATTCTGTCTGCTGTTCAGTGTTTACTTTTTACGAAATCAACTATGGGAGAAGGGGTTGTGAACATTGGTTATTACATTTAGACTGTAGATTGTCATAGTTTCGTGGAAAATAAGTAATCTTTGCCGAAAGCTGTCATGAACAAAGATTAGCTTATCTCAAGTCTGTATTATATAGGGCTTGGCTAGATTTGAGCAACACTTTGAGTTAACTAAACTTTTTGTTTTAATTATAGTAGGCATATGTCTTCAGTTCGTGATTTATAAAAGGCTTGGGATCATGTAGAATGGATAGGGTTATTGTGGTATAATTTGAATAGCATGAAATTCGACCTGTCCTTTTTTAGTTATCTAATTGCACCCGATCTATCGCTTAAAGTATAAGAATATAGTTTTAAGAATTGTCAGTAGCGGGCTTCAAACCCCGTCTCTTCCGCACCCAAGGCGAGCACACTATCAGTTCTGTCCCCAAACACATAAGTATAAATAACGATATACCATCGATAGACAATACAAAAATATAATATTATTCTCTTCCATAATAAAACTTCAGAAATAATAGAATGCGACTTCTTCAATAAAATCATTCATTGTCGTAAGTGGTCGTAAGATAGTAAAACTTGATCTGGAAACCTGAATAAATTTTTAGTTGTGTTGCAGCGAAGAAGTCTGTAGCATGTAGTCCAAGAGATCACGAACTTTGATTTCTGGCCGTTTCTTAAAAACGGCAATGAATGATTACCTCATAACGTGTAGCCAATAGCCTAATGCACGCTTTACCTCTAATAAATCGCCCAATACCTTGAATGCGGGAGCCTTGAATTCAACAAGTTTGTGTAAGCTAATACTTGCCTATACTATTGGCAATCTATAACCACCGCCTAGAAAACTACACGCCTGGCGTTACGAAAATCACCGCTCACCGCATGGTATGATATTGATACTGCGCAGTACATTTTACTCTCTCGACACTTGACAATGTAAAAATGGCCCAAAAGAACCCTTCTCGACCCATGGTAATTAGAATAATATTTTGCAAACAAATAGTCAAATAGACCATTAACGTTTCGCACAAGCCTGATTTCAAGTTAATTTGCTCCGCCAGCAATTTATATTTATCATATGGCTACTTATCAAAAAGTGTCCACAAGGTGGTATATATGACGAAGACATAATTAGGCATTTCCATCCTCTATAACATTACATTAAATTTTAACATTAAGAACATAGGCCTACAGCCCTACACAAAAATAATTTCACTTTTTGAGAGTTGTGTAGGCCTATCTGCCAATTTATACCAATAAATTTGTTTCTATAACATCCGATCATAAAACAGTTTGTTTTGCCAGATCAAGGATTAAATATAAAATAAATGTGTTAATGACATCAGAAATACTACAAGTGATAACGGACGCAAGTCTTGGCCTTGCAAGGGCATTAACGGTGGTCTATAGGATTAATTAACTTGCGTATTTAACCATTTCATTTACACCTCATCAAAACCTGTAGGACTCTTATACACTTCATAGCGATTTCAACTAAAAAATGTTGAAAAGCAATATAAGACACCTTTGACTGGAAACAAAACAATAATGTTATTGTTATAAAGCTGTTGTTTAGATATGTCGAATATTTTCTCAGCTTTTTTCGTTTATTAATATCGTAATACATAAATGGCATTAAAATGTGACAGCTTACTTACCCTCAAACATGAAAACAAAACACAGACTTATTACCATGTTGTTTAACTGCATTGCAAACATATTCACCTGTCGTCTGTCAATTCGCATATGTCATTGTTTGTGTTTGTTACGTCATAATGTTGTAGTGAATACGCTACGGAAACTGAAATTTCGTCGAGCGTGCAATCTAGGCGAGAATAATTAGCGCAACGCAAACAACGTTTCTTAATTAATATCTTTTCCTATTTATCAATTAAATTTTAACAGCTTTTATTTTTTCGCACAATTTTCAATTGTAGGCCTATAAATAGCGAAGTTATATGCAATGAAGTACACTTTTATTTAGTGCATCTTCTCAGTTCAACA3’

Moocci.Sox1/2/3 -1977/-20:

5’TTTTATTTGGGCGTAGAATTGGAAATATTTTATTATTAGAGACGTCTATCAAACTAGTGTTAATTAAAACATATAGTGTCAATAAACATTAATACAGTTTCAAATTTAACCTAGAGTTTCTTTTACGTTACTTTTAAAACGATACGAGCCTTTATATGGTATAATTGTTAAATTTTCTGTAAAAGTTCAAACGTAAAGTTTCTTTTGTAGTTCCTTTATGGGTTGAAATTTATCCCTACCCCTATATATTTGCTTACTAAATGGACATTACCCTACCGTACTTCAAAGTTACCAAATGGGGAAATTACGAATGTAGGCATGACTCAATCGGTTTTATTTTCGTTCGTGAAACTAATGGCCAAAAAATCTGTCAATGAAGAATTTTTTTTGCTTTTACAGCTCATATATTTGTCGTAGTTCACACCGTTACATATGTATATATACTGCTGCACTATGCTGGAATTTTAAAATTTTGTATTTGATGGCAAGAGAGAATAAAGCGTTGGGGAATATCAATGTTTGGATTAAGATTTTCGTTAAATTCATTATTGCCGTTAGGTTATTTATTTTTATAGCAAATCTATGTCAATAAAATTGCAATATACATTAAAATATAATTCATCATAACAGTAAGCGATGAGTGACAATGCGGTTTCACTGGCGTCGGGTTAGAGTAAGGGTGAGGCGGTATTCGTTGACTGACAATTATAACCGTGATATCGTAACGATATTCAATTTTCTTCTCGTATAAAATTGACTTTTGGGAATGTTGCAGGGTAAATTGGTGCGAAAAAAGTAAATATCAATTTATAAAGATATGGATTGTTAGACTCTCGTTAACTTACGTGAGAAGTACTTGATCTCTAACTGAAGAGTGGAAAGTGTTCTGCTCGCCATAGCGTCCCGATGTTAAGACTAACAAACTAGTCCTTGTCTTGGCGAAACTTATGACGTTAAAATCGTCAGTTAAAATATAATCGTTGGCATTACCATTACCCTCTATTCCATTTCATTCCATTCCGACTAAAACACACCCGTTTCGCCAAGGAAAAACACTCGGCCGAAAATAGCTTGCCGGGTTAAGCAGATAGGCGAGACACACTTAAGCAACACCGTAACGTTTCTTCACGCTAAAACTTTCGGCCAGCCCATAAAGCACAGAAATCTTTATTTAGAAATTAGAACGTGTCGAACTTATCATAATGAAATGAGACTATTTAGTTTATCGCTATATTGATCAAATTTTGGAAGCAAAGTTGATAATATATCATAAGATAAGGGTAATATAGTTAATATAGAATATTACGATAAGAAATTTTTGGCTAAGTAACATTCTAAAATATTACGAAGCAAAATTTGCAAGTGATAGCGTAGCTGTTATCGGCAAGGTTCCGCTGGGTATCTCCGTACGCTATTATTTATCGCGTAAGAAAAAATGTGTATCTTTCCATACTCTCATTGCTTGGTTTGTTTAATCAAACTGCGAGCAAAAAAATGCGATGTGTAGAGTAGGATAAAGAGCGAGCGGAGAAGTTATTCAGCGGTAGTTCCAATTGTGAATCATTGTTCAACGGTCACTAGTGTTTAAGTGACAGTGCATCAATAGCTGCGCATTCAAAACGAGAATAGTCTTTGTTAGTTGACGAACAATTGGTCGTTAAATCGTAAGGGTATTGAAGTGTATGTATAGCCAATGTAACATGTAGTACAGTCGGACAATGTTATTGTCTTCTATGAATTTTATTATTTGTTTGAAAATATTTTATAGTGTAGTTTATACAAGTGATAGTAATTTACACAATAGCCGATTATGAAAGTGATAAATATTAATTTCAAATTAATAATAGTTTCGTTTTATTTAGATTTTCTGAAACAATTAAATATTATTCTTAAATTATTGGACAAAGAAAAGTAAAACAATTTAACGAAACAACCTACTCGATCTCAAATCTTCGGGA3’

Moocci.Ebf -3294/+12(isoform “a”):

5’AGACAGCCCATGATGATCTCTTGTTAAATTGTCGAGTTAATCTTGGTTGGATCAAAGTCACGTGACTAATATAGGCTACTTTGTGTTTATAGCTATTATAGATAAACTAGAAACTACTAACAAAGATGAATAAGATTTGGTTTACATCAGGTTTGACATGAATTAAAGCTAATCCGGTAACAAAATTTAAAGCAAACCGATTATCACCTGATTTATTTGTATATAGTTTGGTCACGTGACCAGTGGCGCGTATTTAAAAAATACCGGGTATTCTTGTTACCATACTAAGTGAATTACGTGTTACCATACTAAGTGAATTTACAGATAGAATACAACTGATAACTTCTTGGTAATTTTCAAAGATATTCTTGGTCGGCCAAAAGTCTCCTGACTTATATGGTTCTTTTTGCAATTTACCCCATATCTTATAAAATGCTGCATGGACAGAACGTCATAAAATTTGACATGTATATAGTCCATCAAACTATCTGTCCAAAAAATGAAACTAAAATTTTCAGCGAATTAAAAACAGCAAGGCAAAGTGACAAACGAAATAGCTTGACCTTTAGCCTATAACCTAACCGATTTAGCAAATAATATAAAATGTTTGTTTCAAGTGGTCCAAAATATTTTTTATTCTACAATCAACTTCTATCGATTGAAAGCTAATACTTAAAGAGATGATATAACGAAATCAGGTTTACCATTCAGTTACAGTTATTTAATGAAGTAACTTATGCTTTTCACAGAACAGCAACCTACCCTACATGCTAAACCGATAGGTCTATACCCAGTTTTAAAATTTTGTCTATATGCTATGCATGCCTGTATATACTCTAACCAACCATCTTCATTTTTTATTTATTGCCACATATAATTAGAGATGGCAGTTCATTTATTTTGGGAGAATGTTTAGTATAGAAAAGTGAGCGGAGCGAACATTTATCCACCAACATTGCCACTAAGTGTATTGGCAACCGACCCGCTGCATGCAGAGCGTAGGAATGCACAAAATTAAGACACTCCCTATTTATTATATTTAGTAGATTATAATCTACTAAATATGCCGATGCGTTACGTCGCGCCTTGAACAGCGGTTATCACGGTTTTTATACGTAAACTACTACCACTTAAATAAGGAAATTTATCCGACAGCGTATTGAACTTTCTTAATCGAACAAAGTTTTTCCAAAACATTTCTATAGACATTTAAAATTTTATGCTTAATTTTATGTACCGCATGCTATTTATCCTTTTTACTTTTGCTTAATTTCTAGATCTTTTAATGGCAAGATTTATCACCGTCAGCACCTTGCACTTATTTAAAATTCGTATTATTACTCTTATATTTTTTTTTAATTTATTCGCAGTATTTGGATGTCTTTATGGCGTGTATGGCGTTACTCGTCATTGAAGAGAATATTATGTCGATGGCAAAAACCAGATTAAATAAACTAAACTTGGATAATATATCATTTTTTAATGTGAAAAACGGTGCATGACTATATGTGAGATTAAAAAAAAAGTTTTAGGGATATAATACTGCCCTTGAATTATCCTTTTACAAACTGAACAATTCTACTGTTATAAGAGCTGTTTGTAGTTTTGGATTAGGTATAATCTTAATAAAGTATTTTAAAGCCAGTAGTAATTGGGGAATATTTGTAGTAGGATTAAAAATTTATTATAGTTAAACATAATTTTTCAAATGAACCACGTTAATTTGTCACATACTGTTTGTGATAGAACAAAAAAATATCATTTTAAGCTCAGTTAAAATTCTTTTAAGATATTATGGCTGATCATTTTGTTCAATATCGCGACATATGGGCGGATTCGTATTAATAATGCATTTAAATATCGACCGATTTTGAGCGTTTGCGTTCTCTCGAAATACTCTGAGATTTTTGACTATCATTGTTTTGATACATTATGCTTTCAATCTATTGCCTCGTAATTCTGTTCACTCGGACAGACAAACACACAAACGCTTGATTTTAGAATAGTACAAGCGGATATTTAAAACATGTTTTTTGTTATTGTACACAACTTTTCTATTTAAAGTTGAACACAAAATTTAATAGTTCTCTAATATTTCAGACGTACAAAAAAATTAAATAAAAAAAAAGTGAATATACATAAACAGATTGTAATATTGTTTGATAACGGCTGGATTAATAATTTCAATAACATTGCCATTTTGTATTGAACTCTGAAATCTGGCCAATACACATCAAGTGCATTTAGTGATTACGGACTTGTATTTGTTGTCGCCCTCTTTTATTGTGTATTCAAGAGTATAAACATATTCATTATAAAAATAGTCATCTTTAAATGCTTAAAATTAAAAACTAGAAAGTAATTTTTACTAAGTAAGTACGCTATAATATAGGATATATACACACTAACTTGGATTTGTAGGTAACAGAAAAAAAACATCTATTTTCAAACAGAGTGTATCATAGTCATTAGTATAAAAATGAAGTAAAAGTAAGGATAAAGTAAGTCTCTAAACAATGATAATTCACTAATCGTGTATAAGGATAACTTTTGGTTTCATTGTTAAAAGTAAATTTAAATTTGGTTAATGTTACACGACATAACGTCAGCGAAAAAACGCCATATTGTATTAATTATTGGGTAATTCGTTTCGGGGTCGTGAAATCTTTAACGAGTAAATAGATAAATATAATGTCCCGTCGCCTGATAACAATGGACGCGTGACATTTGTTTATTGTTTATATTTACAGTAAAAACAACATATTGTAGGAAAAGATGGCAGATTTCTTCGTCTGACTCGCAGTTCTACAACGTTGTGTAAAAAGAATTAAACATTCCCTAAAGAAATACTGCTTATAATAAAACAAACAAAAATAAAAAGTCGCGAGAGAAAGTTATGAGATAGTTTTTCATTGGTCGATAGATTTATATCAGATATCTCGAGCGAGTTTTATAAACGTCCCCAACGGTACGGCTGAGTTGATCGAGGTAGGCCCATGGGCATTGCAGATGGTTACTCTTTGATTAGAACCACATCCAACTATACAATCATCTCAGGAGGAATTAAAAATAATGCTTGAAAAATATCATTCAAGTTACGCAAGATGCCCATGCTTGTCCCTCGAGTTCATTGTGACAAAAAACTTGCCGTTGGAAAATGCAGAAAAATTATGTCAGAAAAAATTTTTAAATTAAAGTCAAGATTTAGGTTTTAAAGTTTGCAATAAATACATTTTTGTTTTACAGTTGTTAATCTAATTTAAAGCAATATTTCGGGATAATTAGGTCGATATATGGCGTCTCTT3’

**PROTEIN CODING SEQUENCES**

Predicted START and STOP codons indicated by highlighting.

Primers used to PCR-amplify the sequences from cDNA libraries/clones (or in rare cases, directly from genomic DNA) are underlined.

Moocci.Ets.b full length (VERIFIED SEQUENCE)

5’ATGGAAATGGCGGATCAACATGTTGTGCCAACGTTTGAACAAAGCATCCCTCCTATTGGAATAAAAGAGGAATCAGATTTTTTCGCTGATGAATTTGAAAATGGTGGGAAGAAATTTTCTTATGAAAGTTTATCAACTCCTACTTTTCCATTTGGTACTGCAAATGGAAATTTACCACAAACTGCATTTCCTATGCCTGAAAAATTTTCCCACAAAAAGCAAGCTGAGTGTTTGGATGTTTTGAATGAACAAGAAAACAGAAAAGGATCCATGTTGCAGCTAATGGGTGAACCAGGATTAAACCCAGTATTTAAAATGACTCCGCCAAGAGAGTTGAATACTGTCCCCATTGACAAGGTCAATACGAATATCAAAGATACATCGCCTACTCTAGAGAAATTGTCTAATGGGATTGAAAGTACACAGTTAAAAGATGAGTTAGAAAATGATGCTATGAAAAGTGGAAACAAAAAGAAAAAACCACGACTGTTGTTACGCCTCCCTTCACATGATGATGCAGCTCAGGAAGTACCTACTGGTCAAGAATATGGGACAGAATCAAATGTGCAGTACAATACAAGTGGCTCAAATACACCCGTAGATGAAGTCCCTTCAATGGATAATGTGCTCACGAAGTCAATTGAAGAAGTTCCGAAAACTCCACTTTTTCCTCTTGTTACTTCGGGTACTGGAGCAAAGATCAACGATGCTATCATACAGACATTTGAATCTTTTAAATCTGTCATTAATGAAAAAAGAATGCCTAAGGACCCAAGGCGCTGGCAAGTTGAAGATGTTTTGCAATGGACACGCTGGATTGTTCGGGAGTTCAGCATCCCATCGTTTGACGAAACTAATTTTAGAATGTCAGGCAGTCATCTTTGCGGTTTAAAAAAGGAGGCATTTTTGAAACTTTGCCCCCCATTTGTTGGAGAAATTTTGTGGGAGCATCTGGAACAACTTCAAAAAGATGAAATGGGTTCTACTGAAACAGGTCAGCCTGCTATTCCACCATTCAATTGCAGTATGAACAACAACAAATCGTTGCCATCACATAGCTTCAATGCACCATTGACACCAATCTCACCGCAGTATCCACCAAATCCATCTTATGGAAAGTCAAATCTGCACAGAAGCATGTCTGTGAAACAAGAAACAATGAGTGGTCCGTCCCGCTTCCCCACACACCCACTTACACACTCACATAGCTTTGATAGTGGCAGTGATTGCCAACAAGAACTTGGATGTCATATACCAACACCCCTACCTCCAGTATCTAATCCTTCAGTGTTCAGAAGAAGTTGTTCTCAAGATGAAAGTGGAGGCAAGGTTCAGTTACACACAATGACTCCACTAAGGGAAGAAGAGTCCTCAATGTACCCACACCTTGAACACCTTCACAGATCCCAGTCAAATGTTCCATCCAGTTTGCCATGTGTCAGTAACCAAAGTCGAAGGCCATCTGACATCTCTCACCCATTTCCAACCCCAACCACCCCTAATCTACCTCCATTTCCCCAACCCCAAGATGGAAAATGTGTTACACCATCTGACCCCAGACACCCTCCAGGACCCCAAGATAACTGGCAAGATCTGTATTACGCTAACCTTGCTAATTACCATCACCACCAACATATGGAAAACATCAAGCGTCAGGAAATAGCTCGAGATAAGCTTCATGGTAATATTCATGGAAATCATCCTGTACCTCACCCTGCTTATCCAAATGTTAAGCATGAAAATGTTCCACACCAGGGTTACTGGGGTCCCCAGCAGCACCCACAGTTCCACCAACAGACAATGAAACCCTATGGTATGGAAGAGCGTGCCATGAAGGACCGGCAGTACTTCGGGCCACCAGGAATGTTCCCACCTGGTGGAGAATTTCCAAGCAATCCTGTAATACCTGGAGCTTTCTTAACCGGATATAATGGAAGTGGACCTATTCAACTGTGGCAGTTCTTAATCGAGCTGTTGACTGACAAATCCTGCCAACACTTTGTAACTTGGACTGGGGATGGTTGGGAATTCAAGATGCTCGATCCTGATGAGGTTGCAAGAAGATGGGGAAGAAGAAAAAATAAACCGAAGATGAATTATGAAAAACTATCAAGAGGACTTCGGTATTATTACGACAAAAACATCATTCAAAAGACGGCTGGAAGGCGATATGTTTACCGATTTGTATGCGACCTTCAAAGTCTCCTTGGTTATTCGCCAGCCGAACTACATCAAATGTTGGATGTAAAACCCGAAGATAAAATCACTGGAGATTAA3’

Moocci.Ets.b DBD (DNA-binding domain, to insert in between NLS and VP16/WRPW domain)

5’CCATCCAGTTTGCCATGTGTCAGTAACCAAAGTCGAAGGCCATCTGACATCTCTCACCCATTTCCAACCCCAACCACCCCTAATCTACCTCCATTTCCCCAACCCCAAGATGGAAAATGTGTTACACCATCTGACCCCAGACACCCTCCAGGACCCCAAGATAACTGGCAAGATCTGTATTACGCTAACCTTGCTAATTACCATCACCACCAACATATGGAAAACATCAAGCGTCAGGAAATAGCTCGAGATAAGCTTCATGGTAATATTCATGGAAATCATCCTGTACCTCACCCTGCTTATCCAAATGTTAAGCATGAAAATGTTCCACACCAGGGTTACTGGGGTCCCCAGCAGCACCCACAGTTCCACCAACAGACAATGAAACCCTATGGTATGGAAGAGCGTGCCATGAAGGACCGGCAGTACTTCGGGCCACCAGGAATGTTCCCACCTGGTGGAGAATTTCCAAGCAATCCTGTAATACCTGGAGCTTTCTTAACCGGATATAATGGAAGTGGACCTATTCAACTGTGGCAGTTCTTAATCGAGCTGTTGACTGACAAATCCTGCCAACACTTTGTAACTTGGACTGGGGATGGTTGGGAATTCAAGATGCTCGATCCTGATGAGGTTGCAAGAAGATGGGGAAGAAGAAAAAATAAACCGAAGATGAATTATGAAAAACTATCAAGAGGACTTCGGTATTATTACGACAAAAACATCATTCAAAAGACGGCTGGAAGGCGATATGTTTACCGATTTGTATGCGACCTTCAAAGTCTCCTTGGTTATTCGCCAGCCGAACTACATCAAATGTTGGATGTAAAACCCGAAGATAAAATCACTGGAGAT3’

Moocci.Tbx6-r.a (VERIFIED SEQUENCE)

5’ATGCAGAGTACAGAAATAGCAAACTTTCCGTGGTCTTACGGACAATCTATTACCACCACTGAAACAACTAGCTTTAAAGAAAATGGAGAACATTCAACTGAAGATTTAGTTTCATTAGCGTTTGCAGAAACAGAAAGAAAATCTGAGGAAAATGCTTCTATAATATTTCCAGCTCCTTTCCAGCATCCTTTATTTGACGACAAAAAAGAAGAGAAGGATAATGCGTATATATCCCAGTTTTCTGCTTCACAGCATAATCTTTCAAATTACGACCACGTATCGCCAAATGATCAAAGTTTTAAACCCACAGACATACAACTTGAACTGTGTGACAGAGATCTGTGGGATCAGTTTTCAAAAGTCGGAACAGAAATGATTGTAACAAAAACTGGACGACGAATGTTTCCAGGCTACAGAATAAAAATCACAGGTCTTGACCCGGGTGCAAAATATTGCGTTATGATGGACATCGTAAACGTAGATGACAATCGTTATAAATTTCAGCATGGTGAATGGACAGTTGCTGGAAGAGGAGAACCTCATCTTCCCCAAAGATATTTTCTTCATCCAGATTCACCTGCAACTGGAAATAAATGGATGAAAGAACCTATTTCATTTCATAAAGTAAAACTAACAAACTCTGTAGGACAAGACATAGATGGAAAGGTTGTTTTGAATTCGATGCACCGGTATCAGCCGCGTGTTCATGTGGTCCAATCAGATGACCCATATTCTGTTCACACTCAACCCATGTACACTTTTGCTTTCCCACAAACAGTTTTTATAACCGTAACCGCATACCAAAACGCAGAGGTCACAAAATTAAAAATTGACAACAATCCGTTTGCAAAAGGATTTAGAGATGACGGAGCAAGGTCTAAAAAGAACCGTTGTGAAGGAAACAATTTTCAAAATGACGTTCAGTTTGTTCAACAACAAAAAGTTCAATTGTTACAAAACGAAAAAGTTTCACCACAACCACAATACACTCCATGGAAAACACAGCCATATGTGTACCAGAATTCACAATACAATAGTCATTTTACGAACAATTTTCCACAAAGCTTTTGCAACTGGACCAAGCAACGTTCAGAATTGGAACAGTTCATACCTGTTTATGAAGATCAAATGAATTTTCATTTGCAATATAAACAATGCGAACAATCTTATTACCAAAATAACGTGTCCCCAACAAATATAACATCCACACCTCAAAAACGATTTATTCCTTGTCCACCTGAATCAAACCCTATACCAATGCCAAAGCCGCATTCTCAAGAATACCCACAAATCGAAGCCGCCCAATCAACAGACAGCGGGATTGGTTGGTCCCCACCTACGTCTTCCGACGACGACACGTCATTAATATCCGTTGGAGGACCAGAACAGTTTGAAGGAGTACTTAATAGTAATAACGAAGGAGCTAATAGCGTTCAGTTTGAAAGCAACAACAGTTTTTCGCAAGATTGCAGTTCACTTACAAGCGAAGTTTCTAATCAAGATCCGCAAGACTTTCAAAATTTAGATTGTCCAGATGAGTGGTCTTACTAA3’

Moocci.Tbx6-r.b (VERIFIED SEQUENCE)

5’ATGGAGAATTTAATTTCGATCAACGCTGAGAAATTTAAAAATTTACCGGTACAAGATGTTTACAACAAAGCAGAATGCACGGCTTTACAAGGAGATATAATGGAATACCAGAAAAATTTTAATTCGGTGCAGAATTACAACAGTCCAATCCAAAATGATCAATGGAACACCGATTATCAACACAATTTACCAGAAGCTACGTACGGAAATCAAACTGAAATCCTAAACAGCTACCACGTGGGTGGGATCAACGTGACTTTATGTCAAGCTGACCTATGGCAACAGTTTCATAGAGAAGGAACAGAAATGATTTTGACTAAAAATGGAAGAAGGATTTTTCCTGGATTTCGCATAAAATTAAGCGGACTAAAACCCGACGGATACTATTGCGTTTATCTCGATGCAATTAGTTTAGATAATCATAGATACAAATTTCAAGATGGTGAATGGATGATAGCTGGAAAAAGTGAACCACAACCACCACGCAGATTGGCTTTTCATCCAGATTCTGCATCTCTTGGTAGCGCATGGATGAAAAATACAGTTTCGTTTCATAAAGTTAAGCTGANCAATTCTGTTTCTTGTCGCGATAAAAATGCTTTAATTTTAAATTCTATGCATCGTTATCGACTACGAGTTCATGTAGTAGAATGCTCTGATATTTCCATGGCGCATTTGTTACCTTCGCACTGTACAGTTTTCAATGAAACAAGTTTTGTGACAGTTACTGCTTATCAAAATCATGAAGTTACAAAACTTAAAATTGCAAACAATCCCTATGCTAAGGGTTTTCGAAAAGATGGCGCTAGAGGTTCAAAATCTCTTTCAAGTTCGGATAACGAATATGAGAGTTCTAGCAAACGACCAAATATCTCCTCCTTTACAGGCGGTCATCAGCAATATAATCCACAAGGCAATTCAGTTTACCACCAACCCATTGCTTCGAACTACGGAAACCAGCAACAGCTTTTCCAGCCTTACTATCCACAGCAAACCTACTCAGCCTATAATCAGAATAATGAAAATCAATTTTATCCCGAAATATTATTACCAAATCTCCAAATTCAAAAAGCAGAAACTATTCACCAACCGGCTCCAGCAATAGATAGTGATGAAATATTAGACTTCCTCGATGATTTTCAACCAAGCTCGGCAGAGGTTCTTAGTTCTACAGGAAATGTAGACGCTGTAAAAATGCATACGCCTTTACTCGAACAAGTCACAAATGTATTGGGTTTTGAAAACTTTGACTATACTAAACTTGAAACTATTGAAAACTCAAGATACCCATCTCTAACGGTTCTTGATGAAGGTTATTCTTCGCCAACTTGCTCCAGTTCTCCAGAAAATCATGCGCCAATAAATGGTGTATTTTGATAA3’

Moocci.Lhx3/4.a (VERIFIED SEQUENCE)

5’ATGGAGACAGTTTCTTCGAAATGCCTCGGAGAAAACTTTTACACTCGATATGATGATACAAACAAAATGGCGGACAGTGAAATAGACATATCTCGCGAAAATTCTAATCACATGTTTAATGACGTCACAAGTGCGATGTTTGCAGAGGAATGTGACGACACTGAGGTTTATGAAGATGACATCTTGTTTGATAATGATGAAGTCATTAAAACAAACTTATCGTCGTTTGTTGGAAATCACAAGAAAGATTATTTTTCAACTTCCACGCCGCAAAGAGATAACTTTTCAAAGTGCACTGGTTGTGGACACCACATATTAGACAGGTTCATACTGAAGGTACAGGATAAACCGTGGCACGCAAAGTGTTTAAAGTGCCACGAGTGTTTGTGCCAGTTATCGGAGAAGTGCTACTCCCGAGATAACTATGTTTTTTGCAAAGAAGACTTTTTTAAAAGATTTGGTACGAAATGTGCTGCGTGTGGACAGGGCGTTCCACCTACGGAAGTTGTGCGAAGAGCTCAAGAAAACGTTTATCATCTCGACTGTTTTTGTTGTTTTATTTGCAACGAGAAAATGGACACGGGAGATCATTTCTATTTAATCGAAGACGGAAGACTTGTTTGTAAAAACGATTACGAGCAAGTCAAAGCGAGAGATATTGATTTTGAAAGTGGATCCAAACGACCAAGGACAACAATATCAGCCAAACAGTTGGAAACTTTGAAGTCAGCCTATAACCAAAGTTCGAAACCGGCGCGACATGTGCGCGAACAACTGAGCGCGGATACAGGGCTTGATATGAGGGTGGTCCAGGTTTGGTTTCAAAATCGACGAGCAAAAGAAAAGCGACTAAAAAAAGATGTGGGCAGGCAAAGATGGGGACATTTTTACTCTCAAACTCAAGTCTCAGATGACTCGGTGGATTCAAAACAGGAAATCAAAGACACACAATTTTCCGAACAACAGTCACATTTTATCATAAGTAAAGAAGATATAAGTATCGCATCTCACGAAAACGACATGTACAAGGACAATGAGGGAAGTTTTACGACGTTCAACCCCGATTTCCCCCAGCACTTTCTCTCGCCATCCAACCACCCTGATACTTCTCAATTCCTCGCGTCTGCAGCACTTAACTTGAGAGTTTCCGAAAACGTGTTGCCTCGGCAACACGAAAACTTCACTTTCATTCCGTTTTCTCCCGAAAGCACAAATTCTGAATTTAGTAAGAGTCCAGAATCTTGGTTAGGTGATTTTGATAAAAAACAATCATCGTTTTAGTTTGGACAAACG3’

Moocci.Lhx3/4.b (VERIFIED SEQUENCE)

5’ATGGAATCTTTAATGTTTGAAAGTCCGACAAAGTTCAATTATTTAGTAACAAATGACTCAGGTTACAGCTCTATCCAAAGAACTCCAGACATGTTTACCCAATACTGTGATAATTTCGTAGAATTGGAAGAAATATGTCCCATACCGTTTCCTACACTGAATCAGGATGATGCAGAAGAACCAATACCAGTTAAAGAAACGCAAAGTATTTCGAATATATCCGAGTTGCTTGCAAAGATACCAAAATGTGCAGGATGCGACCTTCATATATTTGATAGTGAATTAATTCAACTCCCAAACCAATCTTGGCATGAAAAATGCATTCAATGTTCCGAATGTTTTTGTCCGTTGGATGAAAATTGTTTTGTTCATGAAAATCTTCTTTTCTGCCGCAACCACTTTTACATGAAATTTGGAGCTGTATGTGCTGCATGCGGCGAAAGAATGTCTCCAAAAGAAATAGTTCGTAGAGCAGAAGAGAAAGTTTATCATGTACAGTGCTTTAAATGTACTCTATGCCAAAAACAAATGGATAACGGAGATTGTTTTTACATAACTAAAGATTGTAGAATAATTTGTAGCCAAGATCACAGTAATTTACAATCTGAAGATTTAGCCGAAATGGAGATTAAACGCCCAAGATCCATAATAACAAACAAACAATTGGACGTTCTCAACTCAATTTACAATCAGAACGCAAAACCACCTCGTCACATCAGGGAGCAACTGAGTCGATCGACTGGTCTCGATATGAGAGTAGTACAAGTTTGGTTTCAAAATAAACGAGCTAAAGAGAAAAGAACAAGAAATACTAACAGAAGAGGCAGTTGGAATCGCTATTAAACA3’

Ciinte.Ets.b (DNA-binding domain used for activator/repressor fusions in this study in yellow)

5’ATGGTAGACCAACATGTTGTACCTTCATCTTTTGATATTAATATTCCCTCAATTAATATTAAGCCTGATGATGATTTCTTTGACAATGATCTCGACTTTGATGTCAAACTCTCCAATGAAGATTTTTCATCACCAGAAGACTTTGTATCGACTCCTGTGTTGCCAGATGACTTTTTACCAGATTTGAACCACTGTAGTATTAAGCAGTCGTCATTCGACAAGCAAATTGCATATGAAAACTTATCAGCACACGATGCAAGCCACATACAGTGCAATGAATCAACAGAGTATTCAAGTTCCTACAACGGCAAACATGAAGTATGTACACCTGTGAAGAACTACCCTGGAAAGGGAAAAGACATTTGCAACATCACACCAAATGTGACTCCAAGATCTGCACAGAAGTCGATGCCAGGCACTCCGCTGGAGGAGCTAACGGGTCTACCTGAGAAACTAGCACCAGAAGATGTGCCAAAAACACCGTTGTTCCCACTTATTACACCTGGTACGGGAGCAAAAATGAACGACGCAATCACTCAGTCATTTTCCTCATTCAGGAATATAATGGAAGCAAACAGCATGTCAAAAGATCCAAAGCTCTGGAGTGCCCCACAAGTGAAAACATGGGCAAGATGGATCGCACAAGAGTTTAGCATCCCATCTTTGGATGAAAGTAATTTCTGCATCTCTGGATCAATGATGTGCAGTCTGAGGAAGGAGAGCTTTTTGCATCTTTGTCCTCCATTTGTGGGCGAGATATTGTGGGAGCATCTTGATAGGTTGCAGTCAGAATGTGGAAATGACACGAGGATACCAGAATGCAGCAACTTAAACCAAAATAACGACAAGATTTCATCATCTACAACACCTCAGAACCCACCCTCCTGCACCACCAACCCACCTTACCCTACTAGACCTGCACCTCCTTACACAAAGAACCCAAGCTTCAACCAACCCATACAAAACCACTTTTCCTTGGACCAACCTCCACAATTCAAAGAAGAAAGATACAGACCCCAACATCTTGATACACCAAGCCATTTCAACCCTACTGATGCCCCTCCTGTTGATATGTCTTGTGTAAAAAGAGAGCAACTTCCACACTACCCAAGCCATGCCTTCCCTGTGAACACAAACCGCCGAAACTTCTCCTTCGAACATTCAATGAACATTAAACCTGAACCAATGTCACACATCAATGCTTTTGACATGCACAGGCAGATGTCGCACCCACACAAGCCAAACCAGCGCTTTGGAATGCATATGGAGAACAGGCGCTCCTCAGAGCCTATACTGACACCTTTGAAGCCGTTCTACAGACACAGACCATTATCCCAGCAGATGTCTCACCCCTTCCCAACCCCTACAACCCCCAACGCACCAGAGCTACCCGGCCACCCCATGCACCTTCCCCCGCATGAAATGGGGGGCCAACAAAACCTTCAAGATCTTTACTACATAAACATAGCAAGTTTACGACATCATCAACAAATTGAAACCATGAAGAGACACGAGATGGCCAAAGGTAAAATTCAACAGACGATCACGAACCGAAGTTTTGGTGACCAACAAGTCAACCAACATCATATGTTATCCCGTAGTGACTCCGTGGGTTGGAATGGCAACAAGGGGAACATTAATGGTCTACTAACCCCTGAACCAGAGGATTTTGAAGGTGGTTTTATGCAACACTCCCGAATGAACAGCTGCATGACACAAAACACAACAGCAGAGTTTCCAAGCAACCCTGTTATACCTGGAGCTTTCCTTACAGGATACAACGGGAGTGGTCCAATCCAACTGTGGCAGTTCCTTATTGAGTTACTGACAGATAGATCTTGCCAACACTTCGTTACATGGACTGGGGATGGCTGGGAGTTTAAGATGATTGACCCAGATGAAGTAGCAAGGAGGTGGGGAAGAAGGAAAAATAAACCTAAGATGAATTATGAGAAACTTTCAAGAGGATTGAGATATTACTATGATAAGAATATCATACAAAAGACAGCTGGAAGGCGTTATGTTTACCGGTTTGTTTGTGATCTTCAAAGTTTGCTGGGTTATTCACCAACAGAACTTCATTCAATGTTGGATGTGAAGCCTGAAGATCGACTAAGTGATGATTAAACAATGTACA3’

**REFERENCES FOR PREVIOUSLY PUBLISHED CIONA SEQUENCES**

Ciinte.Mesp driver: Davidson et al. 2005

CiinteTbx6-r.b driver: Christiaen et al. 2009

Ciinte.FoxD driver: Shi and Levine 2008

Ciinte.FoxF[TVC] driver: Beh et al. 2007

Ciinte.Hand-r driver: Davidson and Levine 2003

Ciinte.Sox1/2/3 driver: Stolfi et al., submitted

Ciinte.Ebf driver: Stolfi and Levine 2011

Ciinte.Tbx6-r.b (coding): Christiaen et al. 2009

Ciinte.Lhx3/4 (coding): Christiaen et al. 2009

Ciinte.Ebf probe: Satou et al. 2002 (*C. intestinalis* Gene Collection Release 1 identifier: 02i14)
